# Polycystic ovary syndrome is transmitted via a transgenerational epigenetic process

**DOI:** 10.1016/j.cmet.2021.01.004

**Published:** 2021-03-02

**Authors:** Nour El Houda Mimouni, Isabel Paiva, Anne-Laure Barbotin, Fatima Ezzahra Timzoura, Damien Plassard, Stephanie Le Gras, Gaetan Ternier, Pascal Pigny, Sophie Catteau-Jonard, Virginie Simon, Vincent Prevot, Anne-Laurence Boutillier, Paolo Giacobini

**Affiliations:** 1 Univ. Lille, Inserm, CHU Lille, Laboratory of Development and Plasticity of the Postnatal Brain, Lille Neuroscience & Cognition, UMR-S1172, FHU 1000 days for health, 59000 Lille, France; 2 Université de Strasbourg, UMR 7364 CNRS, Laboratoire de Neurosciences Cognitives et Adaptatives (LNCA), 12 Rue Goethe, Strasbourg 67000, France; 3 CNRS UMR 7104, INSERM U1258, GenomEast Platform, Institut de Génétique et de Biologie Moléculaire et Cellulaire (IGBMC), Université de Strasbourg, Illkirch, France; 4 CHU Lille, Service de Biochimie et Hormonologie, Centre de Biologie Pathologie, Lille, France; 5 CHU Lille, Service de Gynécologie Médicale, Hôpital Jeanne de Flandre, Lille, France

**Keywords:** PCOS, transgenerational, inheritance, epigenetics, methylation, neuroendocrine, AMH, fertility, metabolic disorder, developmental programming

## Abstract

Polycystic ovary syndrome (PCOS) is the most common reproductive and metabolic disorder affecting women of reproductive age. PCOS has a strong heritable component, but its pathogenesis has been unclear. Here, we performed RNA sequencing and genome-wide DNA methylation profiling of ovarian tissue from control and third-generation PCOS-like mice. We found that DNA hypomethylation regulates key genes associated with PCOS and that several of the differentially methylated genes are also altered in blood samples from women with PCOS compared with healthy controls. Based on this insight, we treated the PCOS mouse model with the methyl group donor S-adenosylmethionine and found that it corrected their transcriptomic, neuroendocrine, and metabolic defects. These findings show that the transmission of PCOS traits to future generations occurs via an altered landscape of DNA methylation and propose methylome markers as a possible diagnostic landmark for the condition, while also identifying potential candidates for epigenetic-based therapy.

## Highlights

• Transmission of PCOS traits in mice occurs via an altered DNA methylation landscape
• Metabolic- and inflammatory-related pathways are dysregulated in models of PCOS
• Common hypomethylation signatures occur in a mouse model of PCOS and in humans
• Identification of a novel epigenetic-based therapeutic strategy for PCOS

## Graphical abstract

**Figure:**
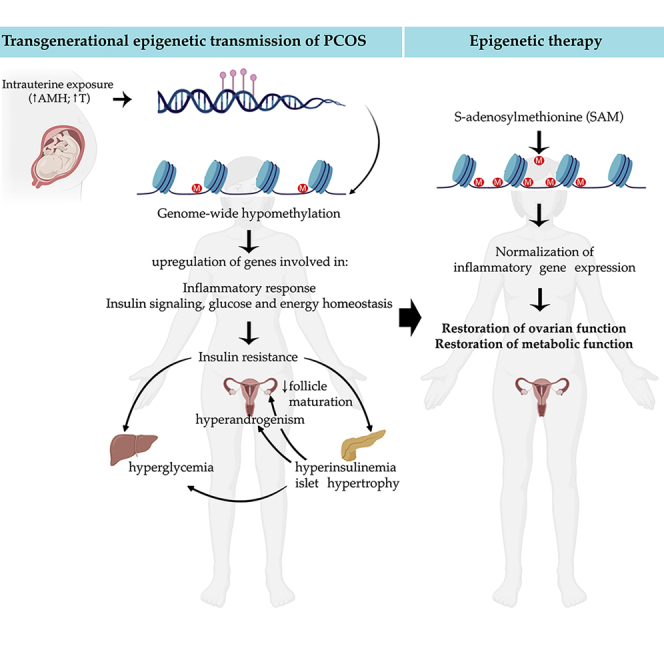

## Teaser

Polycystic ovary syndrome (PCOS) is the most common female reproductive and metabolic disorder, but its pathogenesis is still unknown. Here, Mimouni, Paiva et al. report that in mice, transmission of PCOS-like traits to multiple generations occurs via an altered landscape of DNA methylation and transcriptome expression. These authors also identify methylome markers in women with PCOS as possible diagnostic landmarks and candidates for epigenetic-based therapies.

## Introduction

Polycystic ovary syndrome (PCOS) is the main cause of female infertility, affecting 6%–20% of women of reproductive age worldwide (Dumesic et al., 2015; March et al., 2010). It is characterized by a wide range of clinical symptoms including hyperandrogenism, oligo-anovulation and, in many cases, metabolic disorders (type 2 diabetes [T2D], hypertension, and cardiovascular disease) (Boyle and Teede, 2016; Dokras et al., 2017). Despite the detrimental effects on women’s health, progress toward a cure for PCOS has been hindered by the absence of a clear mechanistic etiology, lack of prognostic markers and by the complexity of the disease.

PCOS has a strong heritable component (Crisosto et al., 2007; Gorsic et al., 2017, 2019), as witnessed by the fact that ∼60%–70% of daughters born to women with PCOS will eventually manifest the disease (Crisosto et al., 2019; Risal et al., 2019). In line with that, daughters of mothers with PCOS have a 5-fold-increased risk of being diagnosed with PCOS later in life (Risal et al., 2019). An altered *in utero* milieu, such as excessive androgen (Abbott et al., 2002; Franks and Berga, 2012; Padmanabhan and Veiga-Lopez, 2013; Risal et al., 2019; Walters et al., 2018), or elevated levels of anti-Müllerian hormone (AMH) exposure (Tata et al., 2018), may be in part responsible for the development of PCOS. Indeed, recent preclinical evidence demonstrated that PCOS may originate in the womb due to the “programing” effect of excessive prenatal AMH exposure (Tata et al., 2018). This animal model, named PAMH, recapitulates all the diagnostic criteria for PCOS in women, including hyperandrogenism, oligo-anovulation, altered fertility, together with increased gonadotropin-releasing hormone (GnRH) and luteinizing hormone (LH) secretion, which exacerbate the hyperandrogenism in mice (Tata et al., 2018) and humans (Stener-Victorin et al., 2020; Walters et al., 2018).

Preclinical PCOS models provide translatable organisms to investigate the mechanisms underlying the etiology of the disease (Stener-Victorin et al., 2020). Consistently, prenatally androgenized (PNA) mice derived from dams exposed to dihydrotestosterone (DHT) during late pregnancy, display PCOS-like phenotypes (Moore et al., 2015; Roland et al., 2010; Sullivan and Moenter, 2004), which are transmitted across three generations (Risal et al., 2019).

Environmental factors exert their effects via the induction of epigenetic changes such as DNA methylation and these modifications can lead to increased disease susceptibility later in life. However, there are very few studies focusing on the epigenetic changes associated with PCOS development, with only a handful of genome-wide studies conducted so far (Makrinou et al., 2020; Shen et al., 2013; Wang et al., 2014; Xu et al., 2010, 2016; Yu et al., 2015). In this study, we provide compelling evidence that PCOS neuroendocrine reproductive and metabolic dysfunctions are transmitted in PAMH mice for at least three generations. We employed genome-wide methylated DNA immunoprecipitation (MeDIP) analysis to characterize methylated genes in ovaries from control and PAMH mice of the third generation, the first unexposed transgenerational offspring, together with transcriptome analysis in these tissues. We identified many genes with altered transcriptome expression in ovarian tissues of PCOS animals and we show that several key molecules associated to the PCOS phenotype are epigenetically regulated through DNA hypomethylation. We report that several differentially methylated signatures found in the ovaries of PCOS-like mice are also present in blood samples from women with PCOS and from daughters born to women with PCOS.

Finally, we provide evidence that treatment of PAMH F3 female offspring with a methylating pharmacological agent rescues the neuroendocrine and metabolic alterations of PCOS, thus highlighting a roadmap to new avenues for epigenetic therapies of the disease.

## Results

### Prenatal AMH treatment drives transgenerational transmission of reproductive and metabolic PCOS alterations across multiple generations

Given the strong heritability of PCOS and the well-documented transmission of the cardinal neuroendocrine and metabolic features observed in first degree relatives of PCOS women (Sir-Petermann et al., 2002, 2012), we sought to test whether female PCOS-like offspring (F1) of gestating mice prenatally exposed to high AMH (F0) (Tata et al., 2018) are susceptible to transfer PCOS-like traits to F2 (intergenerational) and to F3 (transgenerational) offspring.

**Figure 1:**
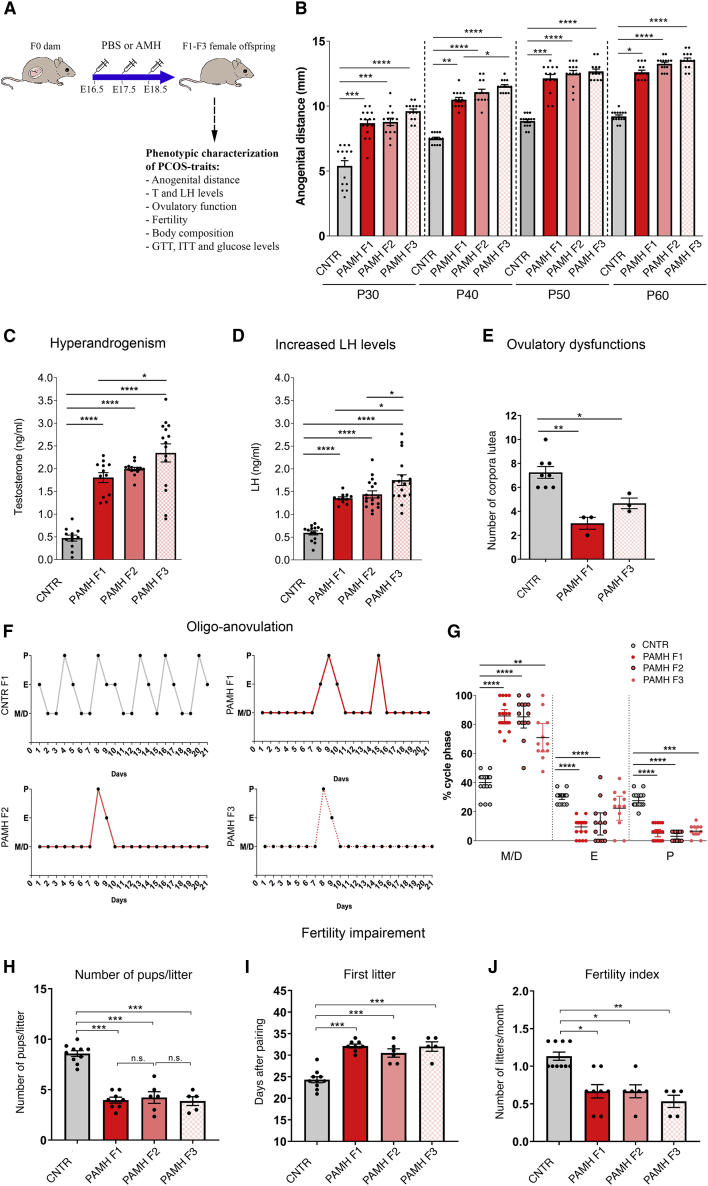
Prenatal AMH exposure induces transgenerational transmission of PCOS neuroendocrine traits to multiple generations (A) Schematic illustration of experimental design employed to generate F1, F2, and F3 offspring. (B) Anogenital distance (AGD) measurement over postnatal days (P) 30, 40, 50, and 60 in adult control females (n = 14), PAMH F1 (n = 13–16), PAMH F2 (n = 14), and PAMH F3 (n = 14). (C) Plasma testosterone concentration in adult females (P60–P90) in diestrus (CNTR F1, n = 12; PAMH F1, n =12; PAMH F2, n = 14; PAMH F3, n = 15). (D) Plasma LH levels in adult (P60–P90) diestrus females (CNTR F1, n = 14; PAMH F1, n = 11; PAMH F2, n = 17; PAMH F3, n = 17). (E) Quantification of the number of corpora lutea (CL) in the ovaries of adult diestrus female mice (CNTR F1, n = 8; PAMH F1, n = 3; PAMH F3, n = 3). (F) Representative estrous cyclicity of 8 mice/treatment group during 16 consecutive days. M/D: metestrus/diestrus phase, P, proestrus; E, estrus. (G) Quantitative analysis of estrous cyclicity in adult (P60–P90) mice from control and PAMH lineages. Scatterplot representing the percentage (%) of time spent in each estrous cycle in CNTR F1 (n = 19), PAMH F1 females (n = 19), PAMH F2 females (n = 14), and PAMH F3 females (n = 12), respectively. The horizontal line in each scatter plot corresponds to the median value. The vertical line represents the 25^th^–75^th^ percentile range. (H) Number of pups per litter. (I) Time to first litter (number of days to first litter after pairing). (J) Fertility index: number of litters per females over 3 months, quantified per generation and pairing. Data in (B)–(E) and (H)–(J) are represented as mean ± SEM. For statistical analysis, p values were calculated by Kruskal-Wallis followed by Dunn's multiple comparisons post hoc test (B and G) or by one-way ANOVA followed Tukey’s multiple comparison post hoc test. ^∗^p < 0.05; ^∗∗^p < 0.005; ^∗∗∗^p < 0.0005; ^∗∗∗∗^p < 0.0001.

We injected pregnant dams (F0) intraperitoneally with PBS (CNTR) or with AMH (AMH_C_, 0.12 mg/Kg/day; prenatal AMH-treated, PAMH) from embryonic day E16.5 to E18.5, to generate CNTR F1 and PAMH F1, respectively. PAMH F1 females mated with PAMH F1 unrelated males generated PAMH F2 offspring and F2 female offspring mated with another group of unrelated males generated F3 offspring (Figure 1A). PAMH F1 female offspring manifest all the major criteria of PCOS diagnosis in humans, including hyperandrogenism, oligo-anovulation, increased LH levels, and fertility impairments (Qi et al., 2019; Tata et al., 2018). We then assessed whether these neuroendocrine reproductive alterations were systematically present in PAMH F2 and F3 offspring. From postnatal day 30 (P30) to P60, F1, F2, and F3 female PAMH lineage exhibited longer anogenital distance than control offspring (Figure 1B), indicating a higher androgenic impregnation in the PAMH lineage. Moreover, PAMH F1-F3 female offspring exhibited delayed vaginal opening and delayed puberty onset (Figures S1A and S1B). At P60, the PAMH lineage did not show any difference in body weight as compared with control females (Figure S1C).

Subsequently, we uncovered a significant and persistent elevation in both circulating levels of testosterone and LH in adult PAMH F1-F3 females in comparison with the control group (Figures 1C and 1D). Ovarian histological analysis of PAMH animals pointed to comparable abnormalities at F1 and F3 consistent with their oligo-ovulatory phenotype, characterized by the presence of fewer post-ovulation *corpora lutea* as compared with control animals (Figure 1E). We confirmed such ovulatory problems by monitoring the estrous cycles of these animals over 3 weeks and showing that F1, F2, and F3 offspring in the PAMH lineage displayed disrupted estrous cycles with prolonged time spent in metestrus and diestrus as compared with the control offspring (Figures 1F and 1G). PAMH lineage also showed diminished fertility and fecundity from F1 to F3, as indicated by fewer pups per litter produced over a 3-month period (Figure 1H), by a significant delay in their first litter (Figure 1I) and by fewer litters produced during the 90 days mating protocol (Figure 1J). Similar ovulatory and fertility defects were detected when PAMH female offspring were mated with control naive males in a matriline breeding scheme (Figures S2A–S2E).

**Figure 2:**
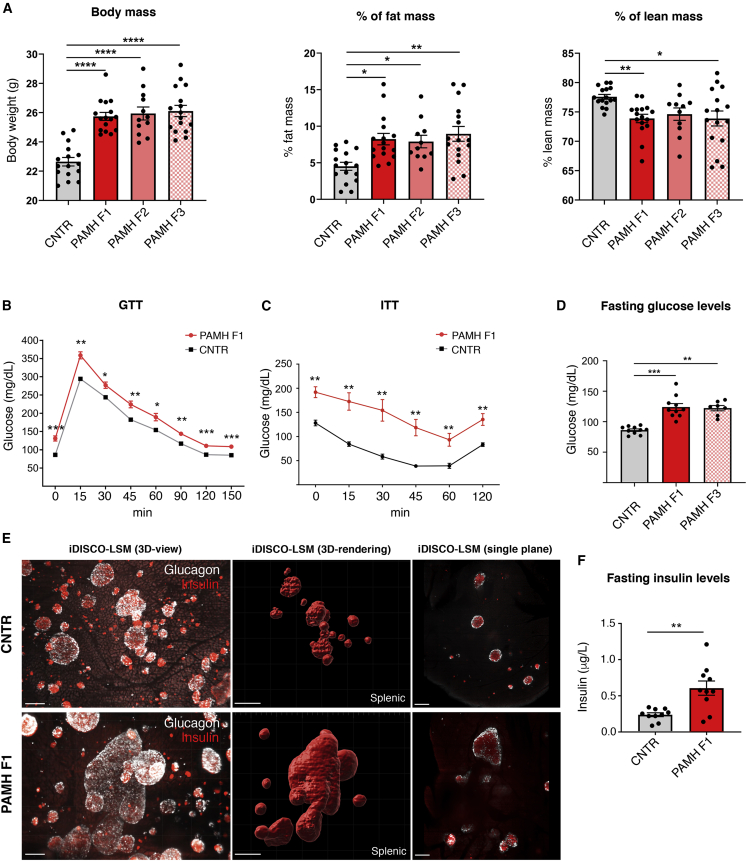
Prenatal AMH exposure causes a transgenerational transmission of metabolic derangements in 6-month-old female offspring (A) Body composition of CNTR (n = 16; 6 months old), PAMH F1 (n = 16; 6 months old), PAMH F2 (n = 11–12; 6 months old), and PAMH F3 (n = 16; 6 months old), presented as body weight (g), percent fat mass normalized to body weight (g), and percent of lean mass. (B and C) Oral glucose tolerance test (GTT) upon 14 h of fasting (B) and insulin tolerance test (ITT) upon 4-h fasting (C) in CNTR (n = 7; 6 months old) and PAMH F1 adult female offspring (n = 7; 6 months old). (D) Glucose levels upon 12 h of fasting in CNTR (n = 10; 6 months old), PAMH F1 (n = 10; 6 months old), and PAMH F3 (n = 7; 6 months old) female offspring. (E) LSFM images of solvent-cleared pancreata dissected from 6-month-old CNTR and PAMH F1 female mice. Left: 3D projection of immunostaining for insulin (red) and glucagon (white); scale bars, 150 μm. Middle: 3D analysis of rendered pancreatic islets expressing insulin; scale bars, 200 μm. Right: single plane optical reslice of the pancreata; scale bars, 200 μm. (F) Plasma insulin levels upon 12 h of fasting in CNTR (n = 10; 6 months old) and PAMH F1 (n = 10; 6 months old) female offspring. Values are represented as the mean ± SEM. For statistical analysis, p values were calculated by one-way ANOVA followed by Tukey’s multiple comparison post hoc test (A, body mass), by Kruskal-Wallis followed by Dunn's multiple comparisons post hoc test (A, % fat mass, % lean mass; D) or by an unpaired two-tailed Student’s t test (B and E). Statistical significance for all analyses were ^∗^p < 0.05; ^∗∗^p < 0.005; ^∗∗∗^p < 0.0005; ^∗∗∗∗^p < 0.0001.

We then checked whether PAMH F1-F3 female offspring presented PCOS-like metabolic alterations. At 6 months of postnatal life, PAMH F1-F3 animals had higher body weight and fat mass, compared with controls (Figure 2A). The percentage of free body fluids was comparable between all groups (Figure 2A). In addition, glucose tolerance and insulin sensitivity were lower in 6-month-old PAMH F1 offspring compared with controls (Figures 2B and 2C).

We then measured fasting glucose levels, upon 12-h overnight fasting conditions, in control and PAMH F1 and F3 female offspring. Fasting glucose levels were significantly higher in PAMH F1 and PAMH F3 animals than in controls (Figure 2D), supporting a hyperglycemic phenotype of these animals. In order to investigate whether PAMH mice displayed a T2D-like phenotype, we first applied a tissue-clearing technique, iDISCO+ (Renier et al., 2014), and whole-organ 3D imaging to determine the 3D distribution of insulin-producing β cells and glucagon-producing α cells, across the whole pancreas in 6-month-old CNTR and PAMH F1 female mice (Figure 2E). Whole-organ immunofluorescence revealed that pancreatic islets of Langerhans were hypertrophic in PCOS-like animals as compared with controls (Figure 2E). To determine whether the islet hyperplasia, observed in PCOS-like animals, was associated with altered insulin secretion, we then measured fasting insulin levels in 6-month-old CNTR and PAMH F1 mice and demonstrated that they are significantly higher in PAMH F1 female offspring than in control animals (Figure 2F).

### Prenatal AMH exposure results in altered ovarian transcriptomic profiles in the third-generation offspring

**Figure 3:**
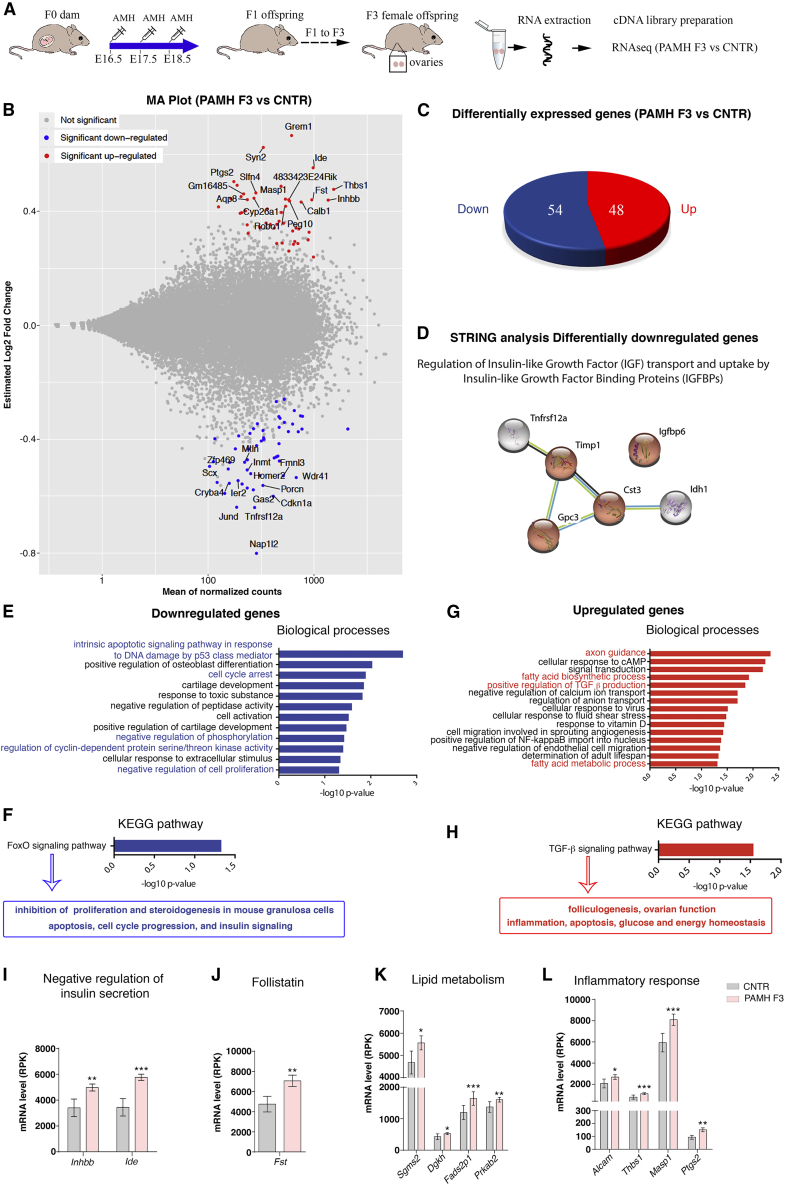
RNA-seq analysis of ovarian tissue in control and F3 PCOS animals points to altered gene expression linked to ovarian and metabolic functions and to inflammatory response (A) Schematic illustration of the experimental design. (B) MA plot of gene expression changes in the PAMH F3 ovaries (n = 4) versus control (prenatally PBS-treated; CNTR, n = 3) for all experimental conditions. Ovaries were dissected from CNTR or PAMH F3 adult females (P60) at diestrus. The MA plot represents the estimated log_2_ fold change as a function of the mean of normalized counts. Significant genes were selected when adjusted p value lower than 0.05. For significant genes, a selection of first gene names according to the adjusted p value is displayed in red for the upregulated genes and blue for the downregulated genes. (C) Pie chart refers to the number of genes upregulated and downregulated when comparing PAMH F3 with CNTR ovaries (p_adj_ ≤ 0.05). (D) STRING protein network analysis of the downregulated genes revealed strong interaction between genes involved in regulating IGF transport and uptake by IGFBPs. (E–H) Functional annotation charts using DAVID performed on the differentially regulated genes either decreased in PAMH F3 versus CNTR (blue, E and F) or increased in PAMH F3 versus CNTR (red, G and H). Significance is indicated as −log10 p value. (I–L) Histograms significantly show enrichment in the PAMH F3 ovaries versus CNTR of genes involved in the negative regulation of insulin secretion (I), follistatin (*Fst*; J), lipid metabolism (K), and inflammatory response (L). ^∗^p_adj_ < 0.05; ^∗∗^p_adj_ < 0.005; ^∗∗∗^p_adj_ < 0.0005.

To dissect the molecular mechanisms and the affected gene pathways underlying “fetal reprogramming” of PCOS, we performed RNA sequencing (RNA-seq) analysis in ovaries dissected from control diestrus offspring (CNTR) and from PAMH F3 diestrus and performed differential gene expression analysis (Figures 3A and S3; Data S1). We identified 102 differentially expressed genes (DEGs; 54 downregulated and 48 upregulated; adjusted p ≤ 0.05) in PAMH F3 ovaries compared with control ovaries (Figures 3B and 3C). Next, we generated heatmaps showing the expression patterns of the 102 DEGs in control and PAMH F3 offspring (Figure S4). Several differentially downregulated genes are involved in regulating insulin-like growth factor (IGF) transport and uptake by IGF-binding proteins (IGFBPs) as shown in the STRING protein interaction network (Figure 3D).

To further gain insight into gene function, we performed a gene enrichment analysis with the DEGs using the database for annotation, visualization, and integrated discovery (DAVID) functional annotation tool (p ≤ 0.05; Figures 3E and 3G). The downregulated-genes-associated biological processes in PAMH F3 offspring are involved in DNA repair, cell-cycle arrest, negative regulation of phosphorylation, and negative regulation of cell proliferation (Figure 3E). We used pathway analyses to identify the significant pathways associated with the DEGs according to Kyoto Encyclopedia of Genes and Genomes (KEGG) (Figure 3F). Our analysis revealed that the most affected pathway is the FoxO signaling pathway, which is related to the regulation of cell cycle and control of quiescence of primordial follicles, steroidogenesis in ovarian granulosa cells, apoptosis, and insulin signaling (Dupont and Scaramuzzi, 2016; Richards and Pangas, 2010). The upregulated genes in the PAMH lineage are involved in axon guidance, fatty acid biosynthetic process, transforming growth factor beta (TGF-β) production, and metabolic processes (Figure 3G). KEGG pathway analysis showed that the most affected pathway among the upregulated genes is the TGF-β signaling pathway, which is involved in folliculogenesis, ovarian function, inflammation, glucose, and energy homeostasis (Dupont and Scaramuzzi, 2016; Richards and Pangas, 2010) (Figure 3H).

Notably, among the upregulated genes, we found some significant enrichment in PAMH F3 offspring of genes involved in the negative regulation of insulin secretion and in the control of folliculogenesis and ovarian steroidogenesis (Findlay, 1993; Poulsen et al., 2020), such as inhibin b (*Inhbb*) and insulin degrading enzyme (*Ide*) (Figure 3I), as well as follistatin (*Fst*; Figure 3J). Moreover, we identified a significant enrichment in the ovaries of PAMH F3 mice of genes involved in lipid metabolism (Figure 3K) and inflammatory response (Figure 3L).

The top 20 significant upregulated and downregulated genes by fold-change in PAMH F3 ovaries versus control ovaries are presented in Figure S5. The top upregulated genes in third-generation PCOS-like ovaries are mainly related to ovarian function, insulin metabolic process, inflammation, angiogenesis, cell-cycle progression, and axon guidance (Figure S5A). The top 20 downregulated genes are mainly linked to epigenetic modifications, such as histone acetylation or methylation, apoptotic process, cell proliferation, and regulation of cell-cycle progression (Figure S5B). Notably, the expression of 7 genes among the top 20 upregulated ones (Figure S5A, asterisks) and 1 gene, among the top 20 downregulated ones (Figure S5B, asterisks), were previously reported to be altered in women with PCOS (Table S1), strengthening the validity of our animal model.

We then validated the RNA-seq results performing qRT-PCR analyses of 6 upregulated genes and 6 downregulated genes related to ovarian function, metabolism, inflammation, axon guidance, and cell migration (Figures S5C and S5D), and we found that the expression of those transcripts is in accordance with the RNA-seq data (Figure S4; Data S1).

### Alterations of DNA methylation patterns in ovaries of PAMH mice

**Figure 4:**
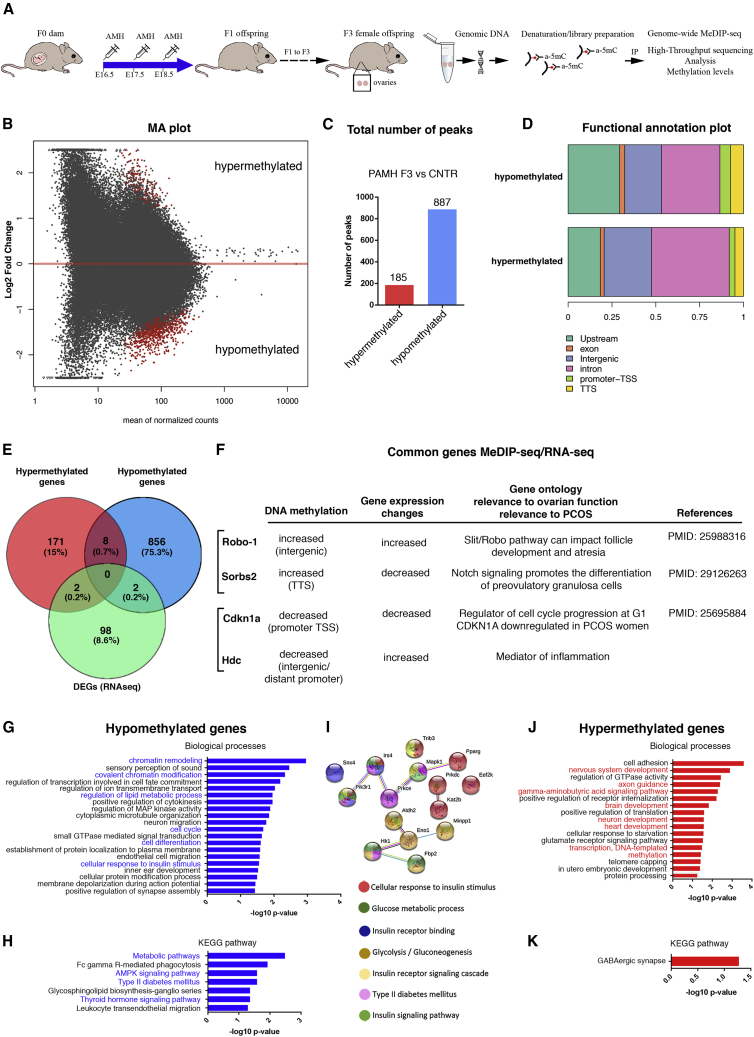
Preponderance of hypomethylations in the ovarian tissue of F3 PCOS animals compared with controls and biological process of hypomethylated and hypermethylated genes (A) Schematic illustration of the experimental design. Methylated DNA immunoprecipitation and deep sequencing (MeDIP-seq) was performed in control ovaries (CNTR n = 3, independent samples at diestrus, P60) and PAMH F3 ovaries (n = 3, independent samples at diestrus, P60). (B) MA plot of MeDIP-seq reads. The MA plot represents the log_2_ fold change as a function of the mean of normalized counts. Log_2_ fold change corresponds to shrunk fold change as calculated with the method proposed in Love et al. (2014). Red dots correspond to significantly different hypermethylated and hypomethylated regions in the ovaries of PAMH F3 animals as compared with controls, adjusted p ≤ 0.05. (C) Total number of methylated regions detected as hyper- or hypomethylated after comparing MeDIP-seq data of the two groups (PAMH3 F3 versus CNTR). (D) Functional annotation plot showing the proportion of methylated regions falling into several genomic features. Upstream refers to upstream regulatory regions. These are regions located −20/−1 kb away from the TSS. Promoter-TSS refers to regions located −1 kb/+100 bp around the TSS and TTS refers to regions located −100 bp/+1 kb around the TTS. Plots are shown for hypomethylated and hypermethylated regions. (E) Venn diagram shows the overlap between genes associated with hypermethylated and hypomethylated regions in ovarian tissues of PAMH F3 mice (MeDIP-seq data) with the 102 DEGs obtained from the RNA-seq analysis. (F) The table shows the list of the 4 common genes found between the MeDIP-seq and the RNA-seq. It details the methylation status, the gene expression changes, and gene ontology related either to ovarian function or PCOS and references when it applies. (G and H) Functional enrichment analysis performed by DAVID on the genes associated with hypomethylated regions when comparing PAMH F3 versus CNTR. GO biological processes (top 20 most significant processes) and KEGG pathway are shown. Significance is indicated as −log10 p value. (I) STRING protein network prediction interaction of proteins associated with glucose metabolism, insulin signaling, insulin response, and insulin receptor binding. (J and K) Functional enrichment analysis performed by DAVID on the genes associated with hypermethylated regions when comparing PAMH F3 versus CNTR. GO biological processes and KEGG pathway are shown. Significance is indicated as −log10 p value.

Since ancestral prenatal AMH exposure leads to alterations in ovarian gene expression in the third generation, we next investigated whether it could modulate the epigenome in PAMH F3 offspring. We used methylated DNA immunoprecipitation (anti-5’methyl-cytosine, 5mC) combined with deep sequencing (MeDIP-seq) to profile the methylomic landscape in control diestrus ovaries (CNTR, prenatally PBS-treated) versus PAMH F3 diestrus ovaries (Figure 4A).

We assessed MeDIP efficiency using spike-in controls for unmethylated and methylated DNA regions from *Arabidopsis thaliana* (Figure S6A). Principal component analysis, particularly the PC2, indicates an evident separation of CNTR and PAMH F3 groups (Figure S6B).

We then calculated the differentially methylated windows between the two groups. Applying an adjusted p value ≤ 0.05 returned 185 significant hypermethylated regions and 887 hypomethylated regions in the ovaries of PAMH F3 offspring as compared with control offspring (Figures 4B and 4C; Data S2), corresponding to 173 exclusively hypermethylated genes and 858 hypomethylated genes.

We defined feature sets spanning sub-typed by location (exon, intergenic, intron, promoter-transcription start site [TSS], and transcription termination site [TTS]) of the hypomethylated and hypermethylated regions (Figure 4D). We also observed that the hypermethylated regions are mostly localized in intronic and intergenic regions, whereas hypomethylated regions are mostly found into upstream-promoters and TSS, thereby most likely affecting gene expression.

To determine whether DNA methylation changes might associate with gene expression variations, we looked for overlap between differentially methylated genes with DEGs of PAMH F3 ovaries (Figure 4E). We identified four common genes between MeDIP-seq and RNA-seq: roundabout homolog 1 (*Robo-1*), sorbin and SH3 domain-containing protein 2 (*Sorbs2*), cyclin-dependent kinase inhibitor 1A (*Cdkn1a*), and histidine decarboxylase (*Hdc*) (Figures 4E and 4F). They are respectively implicated in Slit/Robo pathway, Notch signaling, inhibition of cell proliferation, and inflammation (Figure 4F).

We next performed GO-term enrichment analysis and revealed distinct functional categories for the PAMH-associated gene lists (p ≤ 0.05; Figures 4G–4K). Within the category of biological processes for the hypomethylated genes, chromatin remodeling and chromatin modification, cell cycle, cell differentiation, lipid metabolism, and insulin response are listed in the top related functions (top 20 most significant processes; Figure 4G). Regarding the KEGG pathways, the hypomethylated genes are enriched with metabolic pathways and T2D (Figures 4H and 4I). Genes associated with insulin regulation (glycolysis/gluconeogenesis and T2D) are represented in a STRING protein network predicting interactions of those proteins associated with glucose metabolism, insulin signaling, insulin response, and insulin receptor binding (Figure 4I). Within the category of GO biological processes for the hypermethylated genes, nervous system development, axons guidance, heart development, transcription, and methylation pathways are listed in the top related functions (Figure 4J). KEGG analysis revealed that GABAergic synapse pathway is significantly enriched in the hypermethylated genes (Figure 4K). In accordance with these changes, preclinical investigations in PCOS animal models have reported an ovarian hyperinnervation and a potential contribution of the peripheral sympathetic system in the initiation and/or perpetuation of PCOS has been proposed (Stener-Victorin et al., 2005).

**Figure 5:**
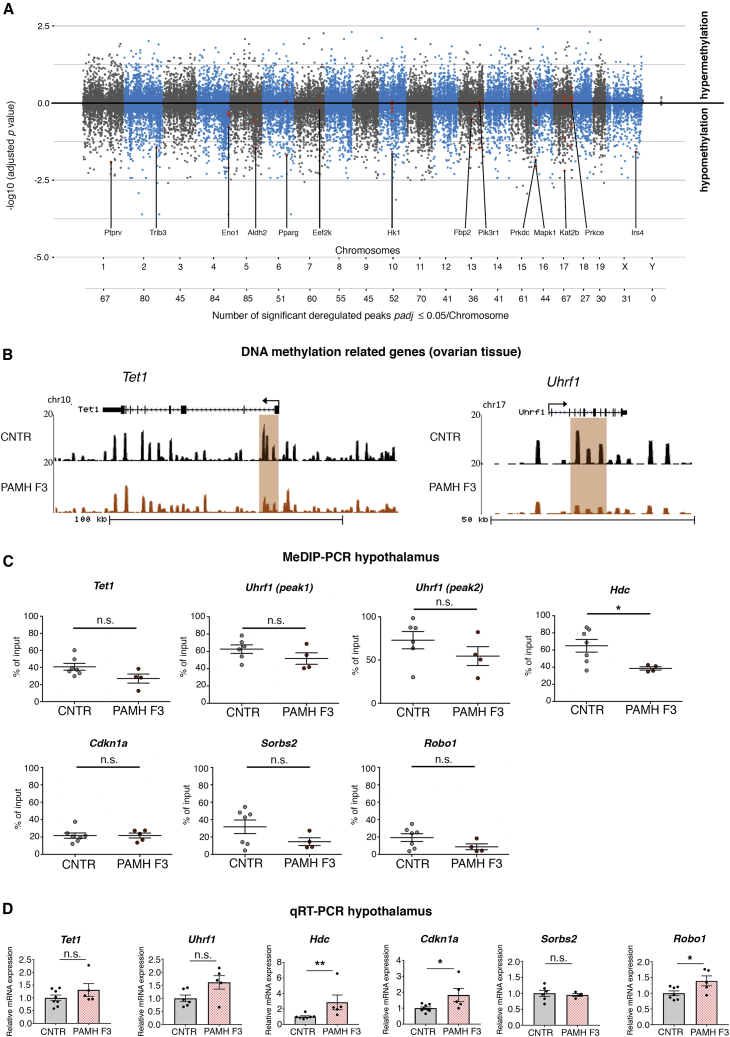
Chromosomal distribution of DNA methylation reads and methylation signatures in the ovary and hypothalamus of PAMH F3 mice (A) Manhattan plot showing the association of methylated positions along the chromosomal positions. x axis represents methylated regions along the chromosomes. y axis is the −log_10_ (adjusted p value), which is the significance of differentially methylated regions when comparing PAMH F3 versus CNTR. Sign of −log_10_ (adjusted p value) corresponds to the direction of methylation change (hyper- or hypomethylated). Red dots show the peaks related to genes associated to hypomethylated regions and whose functional annotations are associated with insulin stimulus, glycolysis/gluconeogenesis and T2D, as depicted in the STRING analysis in Figure 4I. Numbers depicted below the Manhattan plot refer to the total number of significant deregulated peaks with p_adj_ ≤ 0.05 per chromosome. (B) Representative UCSC Genome Browser views of *Tet1* and *Uhrf1* locuses with DNA methylation peaks in ovarian tissues of CNTR versus PAMH F3 mice. Differential methylation analyses revealed that the 5-mC is decreased at the highlighted regions in PAMH F3 mice compared with the CNTR. *Tet1*, p_adj_ = 0.018; *Uhrf1*, p_adj_ = 0.01 (peak 1)/0.02 (peak 2). (C) Genomic DNA was isolated from hypothalami dissected from CNTR (n = 6–7) and PAMH F3 offspring (n = 4–5) and MeDIP-PCR experiments performed in the two groups of animals. Unpaired two-tailed Mann-Whitney U test, ^∗^p < 0.05, n.s. not significant. (D) qRT-PCR analyses using primers against the genes listed were performed in hypothalamic tissues of CNTR (n = 6–8) and PAMH F3 offspring (n = 4–5). Unpaired one-tailed Mann-Whitney U test, ^∗^p < 0.05, ^∗∗^p < 0.005; n.s. not significant. Data in (C) and (D) are presented as mean ± SEM.

Depicting the differentially methylated genes along the chromosomes in a Manhattan plot confirmed a preponderance of hypomethylation in the PAMH F3 samples and indicated that epigenetic changes occur quite homogenously across all chromosomes (Figure 5A). Further, we identified significant changes in DNA methylation in the loci of key genes involved in demethylase activities such as ten-eleven translocation methylcytosine dioxygenase 1 (*Tet1*) and factors responsible for DNA methylation maintenance, such as ubiquitin-like, containing PHD- and RING-finger domains, 1 (*Uhrf1*), in PAMH F3 ovaries compared with control ovaries (Figure 5B). Both the *Tet1* and *Uhrf1* loci are significantly hypomethylated in the third-generation PCOS-like ovaries compared with controls (Figure 5B). Consistent with these findings, we found a higher expression of *Tet1* gene in our RNA-seq analysis of PAMH F3 ovaries versus control ovaries (p = 0.009; Data S1), even though the adjusted p value did not reach statistical significance (p_adj_ = 0.36; Data S1). Overall, these experiments identified many genes and pathways associated with the PCOS reproductive and metabolic phenotypes, with altered DNA methylation profile in ovaries of the third generation of PAMH offspring.

To assess whether changes in DNA methylation detected in ovaries of PAMH F3 animals may also occur in other tissues, we performed MeDIP-PCR experiments in the hypothalamus, as it is the central regulator of both reproductive and metabolic functions, and in the liver, which is responsible for controlling the internal supply of glucose, in CNTR and PAMH F3 animals (Figure 5C). We assessed MeDIP efficiency using primers directed against glyceraldehyde 3-phosphate dehydrogenase (*GAPDH*), as a negative control, and the testicular gene testis-specific histone H2B (*TSH2B*), as a positive control. We found a strong hypomethylation of *GAPDH* and a hypermethylation of *TSH2B*, confirming the efficiency of the immunoprecipitation (Figures S6C and S6D). We screened by MeDIP-PCR the methylation levels of *Tet1*, *Uhrf1*, and of the four common genes between MeDIP-seq and RNA-seq of ovarian tissue: *Hdc*, *Cdkn1a*, *Sorbs2*, and *Robo-1* (Figure 5C). Similar to the methylation changes observed in the ovaries of PCOS-like animals, we found a trend for a hypomethylation in the locus of *Tet1* (p = 0.07; Mann-Whitney test) and a significant hypomethylation in the locus of *Hdc* (p = 0.02; Mann-Whitney test) in the hypothalami of PAMH F3 animals as compared with controls (Figure 5C), whereas we did not detect any methylation changes in the other three genes of CNTR and PCOS animals (Figure 5C). qRT-PCR validation experiments revealed that among those six genes, three (*Hdc*, *Cdkn1a*, and *Robo1*) were significantly upregulated in the hypothalamus of PAMH F3 mice compared with CNTR (Figure 5D). We did not detect any differences in methylation levels in the loci of all the above-mentioned genes in the liver of either animal group (Figure S6F).

### Methyl donor S-adenosylmethionine treatment of PAMH F3 mice normalizes their neuroendocrine reproductive and metabolic phenotypes

**Figure 6:**
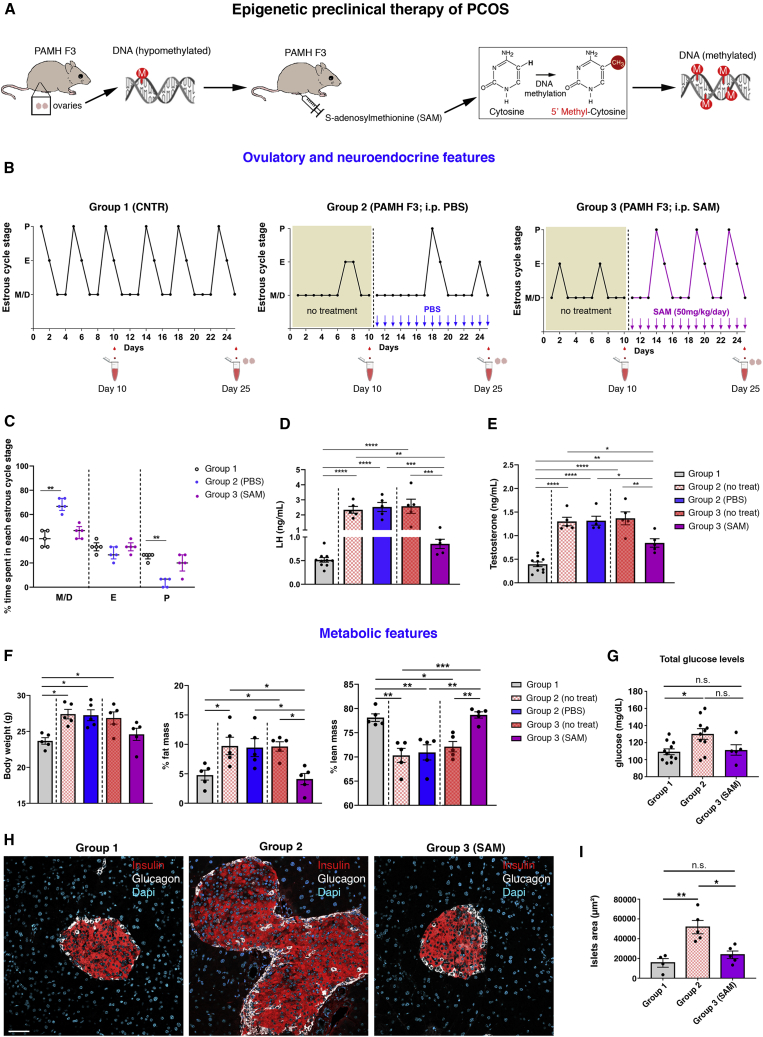
Epigenetic therapy restores PCOS neuroendocrine, reproductive, and metabolic traits in PAMH F3 adult females (A) Schematic of experimental design whereby adult (6 months old) PAMH F3 females have been treated or not with i.p. injections of SAM. SAM functions as the primary methyl donor for transmethylation reactions and acts by adding 5′ methylcytosine groups to the otherwise hypomethylated DNA. (B) Representative estrous cyclicity and experimental design. Prenatally PBS-treated, group 1 (n = 5, 6 months old); PAMH F3 animals, group 2 (n = 5, 6 months old); and SAM-treated, group 3 (n = 5, 6 months old). The y axis refers to the different stages of the estrous cycle: metestrus/diestrus (M/D), estrus (E), and proestrus (P). The x axis represents the time course of the experiments (days). Tail-blood samples were collected for LH and T measurements at day 10 (diestrus), before the beginning of the treatment, and trunk blood was collected at day 25 (diestrus) at the moment of the sacrifice, corresponding to the end of the treatment period. (C) Scatterplot representing the percentage (%) of time spent in each estrous cycle in the three groups of animals, respectively. The horizontal line in each scatter plot corresponds to the median value. The vertical line represents the 25th–75th percentile range. (D) Mean LH levels were measured in diestrus CNTR mice (n = 10) and in group 2 (n = 5) and group 3 (n = 5) before the treatment (day 10) and after the treatment (day 25). (E) Mean T levels measured in diestrus in CNTR mice (n = 10) and in group 2 (n = 5) and group 3 (n = 5) before the treatment (day 10) and after the treatment (day 25). (F) Body composition in the three experimental groups (n = 5 for each group, 6 months old) presented as body weight (grams), percent fat mass normalized to body weight (grams), and percent lean mass normalized to body weight (grams). (G) Mean total glucose levels (mg/dL) in diestrus CNTR mice (n = 11), in PAMH F3 mice (group 2; n = 10), and in PAMH F3 mice after SAM treatment (group 3; n = 5). (H) Representative photomicrographs showing insulin-expressing β cells (red) and glucagon-expressing α cells (white) in the pancreata of CNTR, PAMH F3, and PAMH F3 + SAM mice (females, 6 months old). Scale bar, 50 μm. (I) Quantitative analysis of the mean area of the islets in CNTR (n = 4), PAMH F3 (n = 5), and PAMH F3 after SAM treatment (n = 5). Values in (D)–(G) and (I) are represented as the mean ± SEM. For statistical analysis, p values were calculated by Kruskal-Wallis test followed by Dunn’s multiple comparison post hoc test (C), by one-way ANOVA followed by Tukey’s multiple comparison post hoc test (D, E, G, and I), or by Kruskal-Wallis followed by Dunn's multiple comparisons post hoc test (F). Statistical significance for all analyses was ^∗^p < 0.05; ^∗∗^p < 0.005; ^∗∗∗^p < 0.0005; ^∗∗∗∗^p < 0.0001; n.s. not significant.

Since our MeDIP-seq analyses pointed to a preponderance of hypomethylation in ovarian tissues of PCOS-like animals compared with control animals, we then examined the therapeutic potential of using the universal methyl group donor S-adenosylmethionine (SAM) in an epigenetic preclinical investigation (Figure 6A). SAM is an important and naturally occurring biomolecule found ubiquitously in all living cells and functions as the primary methyl donor for all transmethylation reactions (Bottiglieri, 2002) and can thereby be used to promote methylation of otherwise hypomethylated tissues (Figure 6A). We first analyzed estrous cyclicity of adult control (CNTR; 6-month-old group 1) and PAMH F3 offspring (6 months old) for 25 and 10 days, respectively, to confirm the oligo-anovulatory phenotype of PCOS-like animals (Figure 6B). Thereafter, we monitored for additional 15 days vaginal cytology of PAMH F3 animals treated either with intraperitoneal (i.p.) injections of PBS (group 2) or with 50 mg/kg daily injections of SAM (group 3). We collected tail-blood samples for LH and T measurements at diestrus (day 10), before the beginning of the treatment, and we harvested trunk blood and ovaries at day 25 (diestrus) at the moment of the sacrifice, corresponding to the end of the treatment period (Figure 6B). PAMH F3 animals of group 2 displayed prolonged time in metestrus and diestrus, as compared with control offspring, whereas SAM treatment restored normal ovulation of PCOS animals of group 3 (Figure 6C). In addition, SAM treatment restored the aberrant LH and T concentrations of PAMH F3 offspring (Figures 6D and 6E), as well as their body weight and their body mass composition to control conditions (Figure 6F). Similar to the PAMH F1 animals, the third generation of PCOS-like females were also hyperglycemic (Figure 6G), suggesting that hyperglycemia was transgenerationally passed from F1 to F3 PAMH offspring. The methylating agent lowered total glucose levels of PAMH F3 animals close to control conditions. However, no statistical significance was reached upon 15 days of SAM treatment (Figure 6G). Furthermore, we observed that the pancreatic islet hyperplasia detected in PAMH F1 animals (Figure 2E) was still present in the third generation (Figure 6H) and that it was normalized upon SAM administration (Figures 6H and 6I).

To further explore the effect of SAM on gene expression levels, we harvested the ovaries from CNTR, PAMH F3, and PAMH F3-SAM animals at the end of the treatment period and performed qRT-PCR experiments (Figure S7A). While *Tet1* transcript levels were comparable in the three animal groups, *Uhrf1* was significantly higher in PAMH F3 ovaries (Figure S7A). Notably, SAM treatment restored *Uhrf1* expression in PAMH F3 mice to normal conditions (Figure S7A). We then selected three genes involved in ovarian function and insulin transport that we found both differentially expressed and/or methylated in PAMH F3 offspring versus controls; namely *Sorbs2*, *Grem1*, and *Igfbp6*. Our qRT-PCR experiments confirmed the RNA-seq data and showed a significant downregulation of *Sorbs2* in the ovaries of PCOS animals, while its expression remained unaltered after the SAM treatment (Figure S7A). In agreement with our RNA-seq data, *Grem1* and *Igfbp6* transcripts were higher and lower, respectively, in PAMH F3 mice as compared with controls (Figure S7A). The methylating agent did not affect expression levels of those genes neither (Figure S7A).

As evidence suggests that several metabolic disturbances, including obesity and T2D, are related to the generation of low-grade, chronic inflammation (Reilly and Saltiel, 2017), we next wondered whether a pro-inflammatory state could underlie the metabolic derangements of PAMH F3 mice. We investigated the expression changes in the ovaries of these animals of three genes involved in inflammation and immune response (*Hdc*, *Ptgs2*, and *Rela*). Consistent with our RNA-seq analysis, we identified a 2-fold increase in *Hdc* and *Ptgs2* transcript levels in PAMH F3 ovaries as compared with control animals, which was normalized by the methylating agent (Figure S7A). Similarly, *Rela* (*alias NF-κB*) was upregulated in PCOS-like animals and restored to normal levels upon SAM treatment (Figure S7A).

A positive energy balance is known to initiate an inflammatory response in adipocytes and in hypothalamic centers regulating energy homeostasis (Jais and Brüning, 2017; Reilly and Saltiel, 2017). Thus, we next investigated gene expression changes in visceral fat and hypothalamus of CNTR, PAMH F3, and PAMH F3-SAM animals. We found that *Uhrf1* was significantly overexpressed in PAMH F3 visceral fat compared with CNTR (Figure S7B) and completely restored by the SAM treatment (Figure S7B). Similar to the ovaries of PAMH animals, we found in the visceral fat of PAMH F3 mice a significantly higher transcript expression of inflammatory genes (*Hdc* and *Rela*) and pro-inflammatory cytokines (*Il1b* and *Il6*) as compared with controls (Figure S7B). Among those genes, the epigenetic treatment restored only *Rela* expression levels (Figure S7B). Finally, we found a significant overexpression of *Hdc*, *Ptgs2*, *Il1b*, *Il6*, and *Tnfa* in the hypothalamus of PAMH F3 animals (Figure S7C). The methylating agent normalized the expression of all the inflammatory pathways, except for *Il1b* (Figure S7C).

To determine whether the rescue of LH and T levels observed in PAMH F3 females upon SAM treatment (Figures 6D and 6E) was associated with a central neuroendocrine effect of the methylation agent, we next investigated the expression of two genes regulating proper hypothalamic-pituitary-gonadal (HPG) functions, *Gnrh1* and *Kiss1*, in the hypothalamic preoptic area (POA) dissected from the three groups of animals (Figure S7D). We found a significant upregulation of both transcripts in the POA of PAMH F3 mice compared with CNTR (Figure S7D). However, the methylating agent was not able to restore *Gnrh1* or *Kiss1* levels of PCOS animals to control conditions (Figure S7D).

### Common epigenetic signatures in ovarian tissue of PAMH lineage and blood of women with PCOS

**Figure 7:**
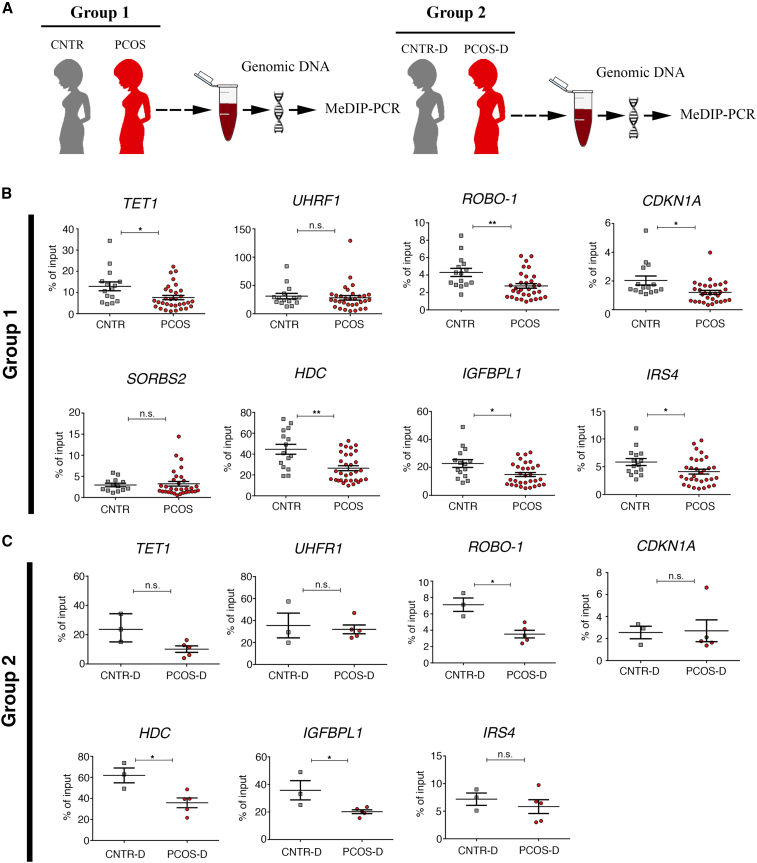
Common epigenetic signatures in human blood samples from women with PCOS (A) Schematic illustration of the experimental design. Genomic DNA was isolated from blood samples of a case-control study comprising two cohorts of women. Group 1: women with and without PCOS (CNTR). Group 2: post-pubertal control daughters born to mothers without PCOS (CNTR-D) and daughters with PCOS born to mothers with PCOS (PCOS-D). Methylated DNA immunoprecipitation using antibody against anti-5mC, followed by PCR (MeDIP-PCR) using specific primers against the genes listed in (B) and (C) was performed in the two groups. (B) MeDIP-PCR analyses in CNTR women (n = 15) and women with PCOS (n = 32). (C) MeDIP-PCR analyses in daughters from the control group (CNTR-D, n = 3) and daughters with PCOS of women with PCOS (PCOS-D, n = 5). Data are presented as mean ± SEM. Unpaired two-tailed Mann-Whitney U test. ^∗^p < 0.05; ^∗∗^p < 0.005; n.s. not significant.

To investigate how our findings in mice might relate to human PCOS, we searched by MeDIP-PCR for common epigenetic signatures in blood samples of women with PCOS and control women (CNTR), as well as in post-pubertal daughters born to mothers with or without PCOS (PCOS-D and CNTR-D; Figure 7A; Tables S2 and S3). We assessed MeDIP efficiency using primers directed against *GAPDH* and *TSH2B* (Figure S6E). Taking into account the hypomethylation of two key DNA methylation related genes in PAMH F3 ovaries, *Tet1* and *Uhrf1*, that could contribute to the preponderance of global DNA hypomethylation identified in PCOS-like mice, we first assessed the methylation levels of *TET1* and *UHRF1* in blood samples of women with PCOS and in healthy women. Interestingly, *TET1* was significantly hypomethylated in PCOS women as compared with controls, whereas methylation levels of *UHRF1* were comparable in the two groups (Figure 7B).

We next selected the four ovarian genes that were differentially expressed and methylated in the PAMH F3 offspring versus controls and investigated the expression of the human equivalent of those genes, *ROBO-1*, *SORBS2*, *CDKN1A*, and *HDC* as well as *IGFBPL1* (encoding for insulin-like growth factor binding protein like 1) and *IRS4* (encoding for insulin receptor substrate 4), which may be related to defects in insulin signaling in PCOS. We identified five (*ROBO-1*, *CDKN1A*, *HDC*, *IGFBPL1*, and *IRS4*) out of the six genes selected from the genome-wide methylation profile of the PAMH lineage as being also differentially methylated in blood samples of women with PCOS as compared with healthy women (Figure 7B). Notably, all changes identified were located in the promoter regions of these genes.

*ROBO-1*, *HDC*, and *IGFBPL1* were also hypomethylated in blood samples of post-pubertal daughters, diagnosed with PCOS, and born to mothers with PCOS as compared with control daughters (Figure 7C). Daughters with PCOS also showed a trend to lower methylation levels of *TET1* as compared with controls (p = 0.07; Figure 7C).

## Discussion

Familial clustering and twin studies point to PCOS as a pathology with a strong heritable component (McAllister et al., 2015). However, the human PCOS loci identified by genome-wide association studies account for less than 10% of heritability (Azziz, 2016), suggesting that environmental and epigenetic mechanisms may play an important role in the etiology of this disease.

Clinical and preclinical studies suggest that altered levels of androgens or AMH during pregnancy may be responsible for the fetal programing of PCOS (Stener-Victorin et al., 2020). Hence, PNA and PAMH animals are excellent preclinical models to mimic a key maternal PCOS condition in which to investigate whether exposed lineages have increased susceptibility to develop PCOS-like traits in F1–F3 offspring (Stener-Victorin et al., 2020). A recent study highlights transgenerational transmission of PCOS-like phenotypes in PNA mice exposed or not to maternal high-fat diet (Risal et al., 2019). That investigation also identifies several genes with altered expression in mouse oocytes from F1–F3 offspring and in the serum of daughters of women with PCOS (Risal et al., 2019). However, the mechanisms underlying the inheritance and transmission of PCOS traits to subsequent generations have not been elucidated. To dissect the cascade of molecular events leading to increased disease susceptibility, we used the PAMH mouse model, which recapitulates the major neuroendocrine reproductive traits of PCOS (Tata et al., 2018).

Compared with the PNA mouse model, in which litter size, estrous cyclicity, and androgen levels of the offspring are unaffected in the second and third generation (Risal et al., 2019), the transmission of PCOS-typical reproductive defects is more severe in the PAMH lineage, which pass on to subsequent generations all the major diagnostic features of PCOS: hyperandrogenism, ovulatory dysfunctions, and altered fertility. This is most likely the result of the different prenatal hormonal exposure protocols used to generate the two mouse models. The PNA model relies on the prenatal non-aromatizable DHT exposure during late gestation while the latter model is derived from exposing the dams to AMH during the same gestational window as the PNA mice (Stener-Victorin et al., 2020). Such treatment drives a 3-fold rise in aromatizable T levels in the dams with subsequent changes in the HPG axis and hormone levels of both the dams and the progeny (Tata et al., 2018). In this study, we also report the appearance of the major metabolic derangements of the human PCOS condition in PAMH mice at six months of postnatal life, including typical traits of T2D and a β cell hyperplasia in the pancreas of PAMH animals of the first and third generation. Islet cell hyperplasia is associated with T2D in the leptin-deficient *ob/ob* mouse, which has been extensively studied as a model for this disease for decades (Bock et al., 2003). Our preclinical data are in line with the observation that up to 30%–40% of women with PCOS have impaired glucose tolerance, and as many as 10% of those women develop T2D by the age of 40 (Diamanti-Kandarakis and Dunaif, 2012; Wild et al., 2010). Consistently, our RNA-seq analysis reveals a significant enrichment in the ovaries of PAMH F3 mice of genes involved in the negative regulation of insulin secretion, including *Inhbb*, *Ide*, *Fst*, and TGF-β signaling pathway, which also regulate folliculogenesis and ovarian steroidogenesis (Findlay, 1993; Liu et al., 2016) and which are affected in human PCOS ovarian tissues (Liu et al., 2016; Pan et al., 2018). Dysfunction of TGF-β signaling in PCOS may be generalized in different organs. Indeed, Dumesic and colleagues (Dumesic et al., 2019) report TGF-β signaling as the “master upstream regulatory gene” in subcutaneous adipose stem cells from women with PCOS in contrast to non-PCOS women. In addition, in PCOS-like, prenatally androgenized female rhesus monkeys, similar disruption of TGF-β signaling accompanies altered DNA methylation patterns of visceral fat, implying an androgen-related developmental origin in PCOS for abdominal fat accumulation (Xu et al., 2011).

Our MeDIP-seq and RNA-seq data show that many affected genes in the ovarian tissue of PCOS animals are correlated with inflammatory and metabolic pathways, typical of the human PCOS condition (Boyle and Teede, 2016; Dumesic et al., 2015; Vázquez-Martínez et al., 2019). Interestingly, alterations in ovarian gene expression patterns, detected in 2-month-old PCOS-like animals, precede the appearance of phenotypic metabolic manifestations in these animals, occurring only few months later, at 6 months. Our preclinical data are also in agreement with several clinical studies reporting DNA methylation changes related to inflammation, hormone-related processes, and glucose and lipid metabolism in various tissues of women with PCOS (Makrinou et al., 2020; Shen et al., 2013; Vázquez-Martínez et al., 2019).

Genetic and epigenetic modifications are implicated in the transgenerational inheritance of prenatally programed diseases (Cavalli and Heard, 2019; Gapp et al., 2014) and in recent years, epigenetic factors gained considerable attention in the study of PCOS (Escobar-Morreale, 2018; Makrinou et al., 2020; Patel, 2018; Vázquez-Martínez et al., 2019). Here, we identify many differentially methylated genes in PAMH F3 ovaries associated with the PCOS phenotype, with a preponderance of hypomethylation detected in these animals. Interestingly, we find that some of the epigenetic signatures and associated gene expression changes detected in the ovaries of PAMH F3 animals are also present in other metabolic tissues, such as the hypothalamus and the visceral fat. These results suggest that altered methylation landscape and transcriptional changes, transgenerationally inherited in PCOS animals, span across multiple tissues involved in the control of ovarian function and metabolism. Such DNA methylation differences are not just general hypomethylation events, but they occur in specific genes and pathways associated with reproductive and metabolic traits associated with PCOS. As we find that all hormonal, reproductive, and metabolic alterations of PAMH F1 offspring are maintained in the third generation, we cannot exclude that AMH fetal-determined changes in epigenetic marks, in combination with the recurring high testosterone levels and perhaps with the metabolic disturbances of the PAMH lineage may be the programing agents responsible for the acquisition and transmission of PCOS-like traits through modifications of DNA methylation landscapes. Also, we cannot exclude a mechanism involving de-methylation and re-methylation that occurred during development in primordial germ cells. Indeed, the maternal genome undergoes passive demethylation before subsequent re-methylation at specific loci to ensure epigenetic inheritance, in which Uhrf1 is a key component (Zeng and Chen, 2019). Our data showing overexpression of *Uhrf1* in PAMH F3 ovary and visceral fat, that is rescued by SAM treatment, support the notion that maintenance of the methylation machinery is a crucial event in the inheritance of PCOS.

We speculate that a global loss of DNA methylation, particularly in promoter-TSS and upstream-promoters, could be responsible for genomic instability in the disease condition. Consistently, a genome-wide DNA methylation study on umbilical cord blood reports a prevalence of hypomethylation in women with PCOS compared with unaffected women (Lambertini et al., 2017). As genomic instability is highly correlated with DNA damage, excessive DNA demethylation could be thus associated with impaired DNA damage repair. This is in line with many reports describing a strong association between PCOS and malignancies, such as ovarian and endometrial cancer (Escobar-Morreale, 2018), and suggest that a higher predisposition to cancer detected in women with PCOS could be due to altered DNA methylation landscapes.

Mechanistically, we identify a significant hypomethylation in the locus of two key genes involved in DNA demethylation (*Tet1*) and DNA methylation maintenance (*Uhrf1*) in PAMH F3 ovaries compared with control ovaries of which our RNA-seq data show increased *Tet1* expression with a significant p value. Consistently, we find *TET1* significantly hypomethylated in blood samples of women with PCOS as compared with control women and a trend to a hypomethylation of this gene is also present in daughters with PCOS. As TET1 is one of the family members of 5mC dioxygenases, which initiate demethylation, it is possible that the decreased levels of *TET1* methylation observed in women with PCOS could contribute to the preponderance of global DNA hypomethylation characterizing the disease.

In this study, we report that only 4 genes showed concordance between RNA-seq and methylation analyses in ovarian tissues of PCOS animals (*Robo-1*, *Sorbs2*, *Cdkn1a*, and *Hdc*). However, a far more complex relationship exists between DNA methylation and transcriptional processes, which can be regulated by other epigenetic mechanisms. Indeed, the methylation of the first exon is negatively correlated to gene expression in a more pronounced way than methylations of the promoter regions (Brenet et al., 2011). Moreover, previous studies suggest a permissive state of gene expression linked with low methylation, but not a linear inhibitory link with high methylation (Anastasiadi et al., 2018). Based on the canonical view of 5mC being a repressor of transcription (Deaton and Bird, 2011), our results indicate a mismatch between the methylation state and the level of gene expression for *Robo-1* and *Cdkn1a*. Considering that the most hypomethylated genes emerging from our analysis are related to chromatin remodeling and chromatin modification, it is likely that, besides DNA methylation, other epigenetic events, such as histone acetylation/methylation are modulated altering gene expression, which could in part explain the weak correlation that we observe between MeDIP-seq and RNA-seq. In line with these findings, histone acetylation alterations are present in various tissues of women with the disease (Qu et al., 2012; Vázquez-Martínez et al., 2019).

Remarkably, we report that several of the differentially methylated genes identified in ovarian tissues of PCOS mice of the third generation are also altered in blood samples from women with PCOS and from daughters of women with PCOS compared with healthy women. Six genes associated with DNA demethylation (*TET1*), axon guidance (*ROBO-1*), inhibition of cell proliferation (*CDKN1A*), inflammation (*HDC*), and insulin signaling (*IGFBPL1*, *IRS4*) are hypomethylated in women with PCOS as compared with controls, and three genes (*ROBO-1*, *HDC*, and *IGFBPL1*) are also hypomethylated in daughters diagnosed with PCOS. As the BMI of women with PCOS included in this investigation is not significantly different compared with controls, both in unrelated women and in CNTR-D and PCOS-D (Tables S2 and S3), and because several metabolic parameters (fasting insulin, fasting glycemia, and triglycerides) are within the normal range in the PCOS groups, we can exclude a priori the contribution of metabolic alterations on the differential methylation landscape of women with PCOS versus control women. We should also note that control women recruited in this study are significantly older than women with PCOS (Table S2), raising the possibility of the existence of an age-dependent epigenetic effect. However, the observation that hypomethylation occurs in daughters with PCOS age-matched with control daughters (Table S3; age CNTR-D: 24 ± 4.4, PCOS-D: 26 ± 2.0), in the same genes as those detected in blood samples of women with PCOS suggests that DNA methylation changes are mostly associated with the PCOS status rather than being influenced by age.

As DNA methylation epigenetic changes can precede phenotypic manifestation and display more stability than gene expression alterations (Kelly et al., 2010), the differentially methylated genes offer the opportunity to develop valuable diagnostic indicators for PCOS risk or prognostic indicators for the disease progression. Even more importantly, the reversible nature of epigenetic modifications makes them more “druggable” than attempts to target or correct defects in gene expression itself. Both hypermethylation and hypomethylation are involved in several disease conditions (Kelly et al., 2010). Several inhibitors of DNA methylation are currently approved for many pathologies by the US Food and Drug Administration (FDA) and have been in clinical use for many years (Kelly et al., 2010). However, at present, there are no FDA-approved therapeutic modalities that target hypomethylation. Here, we examined the therapeutic potential of SAM, a known natural agent causing methylation of several genes (Chik et al., 2014). To our knowledge, this is the first direct evidence for the potential therapeutic effect of SAM in a preclinical model of PCOS. Our investigation showed that SAM treatment can rescue the major PCOS reproductive neuroendocrine and metabolic alterations of PAMH F3 mice, thus highlighting the therapeutic potential of methylating agents as promising epigenetic therapies aimed at treating women with PCOS. We provide evidence that the methylating agent restores the aberrant expression of most inflammatory genes investigated in the ovaries as well as in metabolic tissues of PAMH F3 adult mice. Numerous studies show a causal link between low-grade inflammation and metabolic diseases, including T2D (Reilly and Saltiel, 2017). Moreover, the degree of inflammation correlates well with the severity of insulin resistance, T2D, and hyperandrogenism related to PCOS (González et al., 2006; Zhao et al., 2015). However, the precise triggers of PCOS-associated inflammation are poorly understood. Based on our findings we can speculate that the trigger for tissue inflammations could emanate from altered DNA methylation landscapes, which can be corrected by the SAM.

Taken together, this study points to AMH excess during gestation as a detrimental factor leading to the transgenerational transmission of PCOS cardinal neuroendocrine, reproductive, and metabolic alterations and shed lights into the epigenetic modifications underlying the susceptibility of the disease while pointing to novel diagnostic tools and epigenetic-based therapeutic avenues to treat the disease.

### Limitations of study

In this study, we demonstrate the involvement of epigenetic changes at the ovarian level as drivers of the susceptibility of PCOS across generations. However, we have not performed genome-wide DNA methylation and RNA-seq profiling of the hypothalamic tissue of control and PCOS animals. Hence, we cannot rule out that gene expression modifications and DNA methylation changes possibly affecting the hypothalamus of PCOS-like animals may also play a role in this phenomenon. Moreover, we have not fully assessed if SAM treatment can also normalize the reproductive deficits of PAMH animals.

Future studies, using genome-wide DNA methylation approaches, in combination with preclinical phenotypic investigations, are also necessary to determine whether re-methylation after the epigenetic treatment could potentially drive adverse secondary phenotypic effects at long term. Finally, our clinical investigation identifies methylome markers in the blood of women with PCOS as possible diagnostic landmarks and candidates for epigenetic-based therapies. However, these markers need to be confirmed in larger cohorts of patients, segregating endocrine and metabolic characteristics related to risk and severity of PCOS, to suggest their use as an effective personalized screening and treatment intervention.

## STAR★methods

### Key resources table

**Table:** 

REAGENT or RESOURCE	SOURCE	IDENTIFIER
**Antibodies**		

Mouse monoclonal anti-5′-methylcytosine	Diagenode	Cat#C15200081; RRID: AB_2572207
Mouse IgG	Diagenode	Cat#C15400001; RRID: AB_2722553
Guinea pig polyclonal anti-insulin	DAKO	Cat# IR00261-2; RRID: AB_2800361
Rabbit monoclonal anti-glucagon antibody	Abcam	Cat# ab92517; RRID: AB_10561971

**Biological samples**		

Patient-derived blood	Jeanne de Flandre Hospital, France	http://maternite.chru-lille.fr

**Chemicals, peptides, and recombinant proteins**		

Recombinant human anti-Müllerian hormone (AMH)	R&D Systems	Cat#1737-MS-10
Hematoxylin-eosin	Sigma Aldrich	Cat#GHS132
Trizol	ThermoFisher Scientific	Cat #15596026
S-adenosylmethionine (SAM)	England Biolegends	Cat #B9003S
Dichloromethane	Sigma-Aldrich	Cat# 270997
Benzyl ether	Sigma-Aldrich	Cat# 108014
Methanol	VWR Chemicals	Cat# 20847.360
Hydrogen peroxide solution	Sigma-Aldrich	Cat# 216763
Thimerosal	Sigma-Aldrich	Cat# T8784-5g
Triton X100	Sigma-Aldrich	Cat# X100-500ml

**Critical commercial assays**		

Mouse T ELISA Kit	Demeditec Diagnostics	Cat#DEV9911
RNeasy Lipid Tissue Mini Kit	Qiagen	Cat # 74804
RNA-to-cDNA kit	Applied Biosystems	Cat #4387406
TruSeq Stranded mRNA Library Prep Kit and TruSeq RNA Single Indexes kits A and B	Illumina	Cat # IP-202-1012; IP-202-1024
MagMeDIP kit	Diagenode	Cat # C02010021
Qubit dsDNA HS Assay Kit	Thermo Fisher	Cat # Q32851
QIamp DNA blood Mini kit	Qiagen	Cat # 51104
DNeasy Blood & Tissue Kit	Qiagen	Cat #69504
Mouse Insulin ELISA Kit	Mercodia	Cat #10-1247-01

**Deposited data**		

Raw and analyzed data	This paper	GEO: GSE148839

**Experimental models: organisms/strains**		

C57BL/6J mice	Charles River	N/A

**Oligonucleotides**		

Sorbs2-TTS_forward primer (MeDIP-PCR human samples): CAGCCTCCTGGAGACACTTT	This paper	N/A
Sorbs2-TTS_reverse primer (MeDIP-PCR human samples): CACGTCAAAATGTGGGATCA	This paper	N/A
Robo1_forward primer (MeDIP-PCR human samples): AGGCAAGTTCTGCTCCTCAA	This paper	N/A
Robo1_reverse primer (MeDIP-PCR human samples): TTCTCATTCGCCTGGATTTC	This paper	N/A
Cdkn1a-prom_forward primer (MeDIP-PCR human samples): GCAGAGAGGTGCATCGTTTT	This paper	N/A
Cdkn1a-prom_reverse primer (MeDIP-PCR human samples): TCTGGCAGGCAAGGATTTAC	This paper	N/A
Hdc-inter_forward primer (MeDIP-PCR human samples): CCTGCCCAATTCAATCTGTT	This paper	N/A
Hdc-inter_reverse primer (MeDIP-PCR human samples): CCGGAATAGGGTGGTAGTCA	This paper	N/A
Igfbpl1-prom_forward primer (MeDIP-PCR human samples): CACCCCTCATGTGTCTTGTG	This paper	N/A
Igfbpl1-prom_reverse primer (MeDIP-PCR human samples): CCAAGAACGGTGTAGCTGGT	This paper	N/A
Irs4-prom_forward primer (MeDIP-PCR human samples): TAACCAGTGTTGGGCTGTGA	This paper	N/A
Irs4-prom_reverse primer (MeDIP-PCR human samples): TCTCGCTCAAGGAAAGGAAA	This paper	N/A
Uhrf1_forward primer (MeDIP-PCR human samples): TCTTCCGGGTTTGTCATCTC	This paper	N/A
Uhrf1_reverse primer (MeDIP-PCR human samples): CGAACTTGGCAGGTAGGAAG	This paper	N/A
Tet1-prom_forward primer (MeDIP-PCR human samples): GAGGGCTCTCACACTTCCTG	This paper	N/A
Tet1-prom_reverse primer (MeDIP-PCR human samples): GCTCAGTTTCCTCCAGCAAC	This paper	N/A
Sorbs2 _forward primer (MeDIP-PCR mouse samples): CTATCATCATTGCGGGTTCA	This paper	N/A
Sorbs2_reverse primer (MeDIP-PCR mouse samples): ATGGGACAACCTGACACACA	This paper	N/A
Robo1_forward primer (MeDIP-PCR mouse samples): GCAAAGCTCTCTGCTTTTGAA	This paper	N/A
Robo1_reverse primer (MeDIP-PCR mouse samples): AACGTGTCACTCCCTTCTCC	This paper	N/A
Cdkn1a _forward primer (MeDIP-PCR mouse samples): GTATGCTGCCACAACCACAC	This paper	N/A
Cdkn1a_reverse primer (MeDIP-PCR mouse samples): GCTGCTACTTGGCACCAGTT	This paper	N/A
Hdc _forward primer (MeDIP-PCR mouse samples): GAACGTCATTCTCACAGAGCA	This paper	N/A
Hdc_reverse primer (MeDIP-PCR mouse samples): CCCATTCTCACCACCGATTA	This paper	N/A
Uhrf1-peak1_forward primer (MeDIP-PCR mouse samples): TCTTCCCTGGGCTTCCTAGT	This paper	N/A
Uhrf1-peak1_reverse primer (MeDIP-PCR mouse samples): AGCGAGCTTCACACACACAG	This paper	N/A
Uhrf1-peak2_forward primer (MeDIP-PCR mouse samples): TTTGTGGGTCTGACAACTGG	This paper	N/A
Uhrf1-peak2_reverse primer (MeDIP-PCR mouse samples): TGGAAGGCATATGCTCAGTG	This paper	N/A
Tet1_forward primer (MeDIP-PCR mouse samples): TGGGATCCTATGTCCTCTTCC	This paper	N/A
Tet1_reverse primer (MeDIP-PCR mouse samples): TCCACCTGCCTCTACCTTTTT	This paper	N/A
Gremlin 1	TermoFisher Scientific	Cat#Mm00488615_s1
Follistatin	TermoFisher Scientific	Cat#Mm00514982_m1
Roundabout homolog 1 (Drosophila)	TermoFisher Scientific	Cat#Mm00803879_m1
insulin degrading enzyme	TermoFisher Scientific	Cat#Mm00473077_m1
prostaglandin-endoperoxide synthase 2	TermoFisher Scientific	Cat#Mm00478374_m1
insulin-like growth factor binding protein 6	TermoFisher Scientific	Cat#Mm00599696_m1
ephrin B1	TermoFisher Scientific	Cat#Mm00438666_m1
tumor necrosis factor receptor superfamily, member 12a	TermoFisher Scientific	Cat#Mm01302476_g1
Jun D proto-oncogene	TermoFisher Scientific	Cat#Mm04208316_s1
cyclin-dependent kinase inhibitor 1A (P21)	TermoFisher Scientific	Cat#Mm04205640_g1
growth arrest specific 2	TermoFisher Scientific	Cat#Mm00433519_m1
aquaporin 8	TermoFisher Scientific	Cat#Mm01278161_m1
actin, beta	TermoFisher Scientific	Cat#Mm00607939_s1
ubiquitin-like, containing PHD and RING finger domains, 1	TermoFisher Scientific	Cat#Mm00477872_m1
tet methylcytosine dioxygenase 1	TermoFisher Scientific	Cat#Mm01169087_m1
sorbin and SH3 domain containing2	TermoFisher Scientific	Cat#Mm01200787_m1
histidine decarboxylase	TermoFisher Scientific	Cat#Mm00456104_m1
nuclear factor of kappa light polypeptide gene enhancer in B cells	TermoFisher Scientific	Cat#Mm00476361_m1
mannan-binding lectin serine peptidase 1	TermoFisher Scientific	Cat#Mm00434830_m1
allograft inflammatory factor 1	TermoFisher Scientific	Cat#Mm00479862_g1
interleukin 6	TermoFisher Scientific	Cat#Mm00446190_m1
tumor necrosis factor alpha	TermoFisher Scientific	Cat#Mm00443258_m1
interleukin 1 beta	TermoFisher Scientific	Cat#Mm00434228_m1
nuclear factor of kappa light polypeptide gene enhancer in B cells 1	TermoFisher Scientific	Cat#Mm00476361_m1
Kisspeptin 1	N/A	Cat#Mm03058560_m1
gonadotropin releasing hormone 1	N/A	Cat#Mm01315605_m1
glyceraldehyde-3-phosphate dehydrogenase	N/A	Cat#Mm99999915_g1

**Software and algorithms**		

Prism 8 Version 8.3.1	GraphPad Software, LLC	https://www.graphpad.com/scientific-software/prism/
Inspector software	LaVision Biotec	http://www.lavisionbiotec.com/
Imaris x64 software (version 8.0.1)	Bitplane	http://www.bitplane.com/imaris/imaris
iMovie (version 10.1.1)	Apple	http://www.apple.com/fr/imovie/

**Other**		

MiniSpec mq 7.5, Nuclear Magnetic Resonance	Bruker	N/A
Glucometer	Life scan	OneTouch Verio
Bioruptor Plus sonicator	Diagenode	Cat # B01020001

### Resource availability

#### Lead contact

Further information and requests for resources and reagents should be directed to and will be fulfilled by the Lead Contact, Paolo Giacobini (paolo.giacobini@inserm.fr).

#### Materials availability

This study did not generate new unique reagents.

#### Data and code availability

The accession number for the RNA-seq and MeDIPseq data from mouse ovaries from CNTR and PAMH F3 females reported in this paper is GEO: GSE148839. All the other data supporting the findings of this study are available within the article and its supplementary information files and from the lead contact author upon request.

### Experimental model and subject details

#### Human studies

Blood samples have been collected prospectively, from 2003 to 2008, to perform genetic studies, in the Reproductive Medicine Department of Jeanne de Flandre in Lille University Hospital, France. Biological and clinical data about patients were collected at the same time. This study was approved by the Ethics Committee of Lille University Hospital (DRC BT/JR/DS/N° 0231 PROM 02-563 CP 03/11). Written informed consent was obtained for all patients. Patients were initially referred to our department for hyperandrogenism (HA) and/or oligo-anovulation and/or infertility. The diagnosis of PCOS was based on the presence of at least 2 out of the 3 following Rotterdam criteria (Rotterdam, 2004), i.e.,: −1) HA (clinical or biological). Clinical HA was defined by the presence of hirsutism (modified Ferriman-Gallwey score over 7 and/or acne located in more than two areas). Hyperandrogenism was defined as a serum TT level > 0,39 ng/ml and/or a serum androstenedione level (A) >2,2 ng/ml, as previously reported (Dewailly et al., 2011) −2) oligo-anovulation, (i.e. oligomenorrhea or amenorrhea); −3) presence of Polycystic Ovarian Morphology (PCOM) at ultrasound (U/S), with an ovarian area ≥ 5.5 cm^2^ and/or a follicle number per ovary ≥ 12, unilaterally or bilaterally. Women with congenital adrenal hyperplasia, Cushing syndrome, androgen secreting tumor or hyperprolactinemia were excluded. Women with PCOS were asked about familial history and the genetic study was also proposed to their mothers and sisters. The latter were asked about their personal clinical history (age, body mass index, age of first menstruations, cycle length, presence of hirsutism or acne). For sisters who didn’t have any contraceptive treatment, hormonal assays were also performed in the follicular phase. Based on this information, they were classified as women with PCOS or control, if possible.

Biochemical and hormonal measurement tests were performed in the central biochemistry department of Lille and included: estradiol, LH and FSH, total testosterone, delta4 androstenedione, 17-hydroxyprogesterone, SDHEA, SBP, prolactinemia, fasting glucose, insulinemia and lipid profile. Estradiol, androstenedione, testosterone, LH and FSH were measured by immunoassays as previously described (Dewailly et al., 2011; Pigny et al., 1997). Fasting serum insulin levels were measured in duplicate by an immunoradiometric assay (Bi-Insulin IRMA Pasteur, Bio-Rad, Marnes la Coquette, France) that uses two monoclonal anti-insulin antibodies. Intra and interassay coefficient of variation were <3,8 and <7,5% respectively. Results are expressed as milli international units per liter.

47 blood samples have been recently analyzed from 32 women with PCOS (18-65 years old) and 15 women without PCOS (22-66 years old). Among the 32 PCOS women, five were born from PCOS mothers (23-30 years old) and among the 15 control women, 3 were confirmed to be born from control mothers (22-36 years old).

All procedures contributing to this work comply with the ethical standards of the relevant national and institutional committees on human experimentation and with the 1975 Declaration of Helsinki, as revised in 2008.

#### Mouse models

All C57BL/6J (B6) mice (Charles River, USA) were group-housed under specific pathogen-free conditions in a temperature-controlled room (21-22°C) with a 12-h light/dark cycle and *ad libitum* access to food and water. Standard diet (9.5 mm Pelleted RM3, Special Diets Services, France) was given to all mice during breeding, lactation and growth of young stock. Nutritional profile of the standard diet RM3 is the following: Protein 22.45%, Fat 4.2%, Fiber 4.42%, Ash 8%, Moisture 10%, Nitrogen free extract 50.4%; Calories: 3.6 kcal/gr. F0 C57BL/6J mice were used directly after arrival into the animal facility and after an acclimatization period of at least 2 weeks.

Mice were randomly assigned to groups at the time of purchase or weaning to minimize any potential bias. No data sets were excluded from analyses. The animals were daily for health issues by qualified personnel; health status was normal for all animals.

Animal studies were approved by the Institutional Ethics Committees of Care and Use of Experimental Animals of the University of Lille (France; Ethical protocol number: APAFIS#2617-2015110517317420 v5 and APAFIS#13387-2017122712209790 v9). All experiments were performed in accordance with the guidelines for animal use specified by the European Council Directive of 22 September 2010 (2010/63/EU). The sample size, sex and age of the animals used is specified in the text and/or figure legends.

### Method details

#### Prenatal anti-Müllerian hormone (PAMH) treatment

PAMH animals have been generated as previously described (Tata et al., 2018). Timed-pregnant adult (3-4 months) C57BL6/J (B6) dams were injected daily intraperitoneally (i.p.) from embryonic day (E) 16.5 to 18.5 with 200 μL of a solution containing respectively: 1) 0.01 M phosphate buffered saline (PBS, pH 7.4, prenatal control-treated, CNTR), 2) PBS with 0.12 mgKg^-1^/d human anti-Müllerian hormone (AMH) (AMH_C_, R&D Systems, rhMIS 1737-MS-10, prenatal AMH (PAMH)-treated).

#### Mouse breeding scheme and feeding paradigm to generate F1–F3 offspring

PAMH female offspring (F1) were mated with F1 PAMH unrelated males to generate PAMH F2 offspring, and a subset of PAMH F2 female offspring were mated with PAMH F2 unrelated males to generate PAMH F3 offspring. The remaining F1, F2 and F3 female offspring were subjected to phenotypic testing as described below. Control male or female offspring (CNTR) used in this study were generated by prenatally treating gestating mice with PBS from E16.5 to E18.5 as described above.

The exact number of mice used for each procedure and their sex and age are given in the figure legends and/or text. Details of the number of mice used for (1) phenotypic testing and (2) breeding to generate F1, F2 and F3 offspring in each group are specified in in the figure legends and/or text. To ensure variability within each group, offspring in each generation were randomly allocated for phenotypic testing or breeding.

#### Assessment of phenotype, estrous cycle, and fertility

Control F1 and PAMH F1-F3 female offspring were weaned at post-natal day P21 and checked for vaginal opening (VO) and time of first estrus. Anogenital distance (AGD) and body mass (grams, g) were measured at different ages during post-natal development (P30, 35, 40, 50 and 60). At VO and in adulthood (P60), vaginal smears were performed daily for 16 consecutive days (4-cycles) for analysis of age of first estrus and estrous cyclicity. Vaginal cytology was analyzed under an inverted microscope to identify the specific stage of the estrous cycle. The reproductive competency of these animals was determined by pairing the following mice: CNTR F1 females mated with CNTR F1 males, CNTR F1 males mated with PAMH F1-F3 females, PAMH F1-F3 females mated with PAMH F1-F3 males, for a period of 3 months. Unexperienced males and primiparous females, selected from at least three different litters, were used for the 90-days mating protocol test. Number of pups/litter (number of pups), fertility index (number of litters per females over 3 months), and time to first litter (number of days to first litter after pairing) were quantified per treatment and pairing.

#### Ovarian histology

Ovaries were collected from 3-month-old diestrus mice, immersion-fixed in 4% PFA solution and stored at 4°C. Paraffin-embedded ovaries were sectioned at a thickness of 5 μm (histology facility, University of Lille 2, France) and stained with hematoxylin-eosin (Sigma Aldrich, Cat # GHS132, HT1103128). Sections were examined throughout the ovary. Total numbers of corpora lutea (CL) were classified and quantified as previously reported (Caldwell et al., 2017). To avoid repetitive counting, each follicle was only counted in the section where the oocyte’s nucleolus was visible. To avoid repetitive counting, CL were counted every 100 μm by comparing the section with the preceding and following sections. CL were characterized by a still present central cavity, filled with blood and follicular fluid remnants or by prominent polyhedral to round luteal cells.

#### LH and T ELISA assays

LH levels were determined by a sandwich ELISA, using a well-established ELISA method (Steyn et al., 2013; Tata et al., 2018). A mouse LH-RP reference was provided by Albert F. Parlow (National Hormone and Pituitary Program, Torrance, California, USA). The assay sensitivity of the LH ELISA was 0.04 ng/ml and intra-assay coefficient of variation was 4.3%. Plasma T levels were analyzed using a commercial ELISA (Demeditec Diagnostics, GmnH, DEV9911) according to the manufacturers’ instructions as previously validated in other studies (Moore et al., 2015; Tata et al., 2018). The assay sensitivity is 0.066 ng/ml at the 2 standard deviation confidence limit. Intra-assay coefficient of variation for testosterone was 7 % and inter-assay coefficient of variation was 11.0%. 20 μl of plasma was used for the testosterone assay, run in duplicate samples.

As T and LH levels can vary significantly due to handling, sampling techniques and depending on the time of the day, female adult mice were habituated with daily handling for 3 weeks. Blood samples were taken from trunk blood, for T measurements, and from the tail, for LH measurements, between 10h00 and 12h00 during diestrus.

#### Body weight and composition

Whole body fat, fluids, and lean tissue mass were determined by Nuclear Magnetic Resonance. (MiniSpec mq 7.5, RMN Analyser, Bruker) according to the manufacturer’s recommendations.

#### Measurement of fasting blood glucose and insulin level

Fasting blood glucose levels were assessed after animals were fasted for 12 h (starting from 8:00 PM). Blood glucose levels were determined in blood samples from the tail vein at 8:00 AM using an automatic glucometer (OneTouch Verio, Life scan). Fasting Insulin levels were determined in plasma samples after sacrifice, by a sandwich Insulin ELISA (Mercodia, Cat #10-1247-01) according to the manufacturers’ instructions.

#### Glucose and insulin tolerance tests

For intraperitoneal glucose tolerance test (ipGTT), animals were subjected to an overnight fasting (14 h food withdrawal). For intraperitoneal insulin tolerance test (ipITT), mice were fasted for 4 h. Either glucose (2 g/kg body weight) or human normal insulin (0.75 U/kg body weight) were injected intraperitoneally at 0 (prior to glucose or insulin administration) and blood was collected from the tail vein at different time points (0, 15, 30, 45, 60, 120, 150). Plasma glucose was measured using an automatic glucometer (OneTouch Verio, Life scan).

#### Optical clearing of mouse pancreas

Whole-organ staining and clearing were performed using iDISCO+ (Renier et al., 2014). Pancreata were dissected from perfused animals and processed as described below. Samples were dehydrated [20, 40, 60, 80, and 100% methanol at room temperature (RT)], delipidated [100% dichloromethane (DCM; Sigma-Aldrich)], and bleached in 5% H2O2 (overnight, 4°C). Pancreata were rehydrated (80, 60, 40, and 20% methanol) and permeabilized [PBS/0.2% TritonX-100 (twice for 1h)] before proceeding to the staining procedures. Samples were incubated at 37°C on an adjustable rotator in 10 ml of a blocking solution (PBSGNaT) of 1X PBS containing 0.2% gelatin (Sigma-Aldrich), 0.5% Triton X-100 (Sigma-Aldrich) and 0.01% NaAzide for 3 nights. Samples were transferred to 10 ml of PBSGNaT containing primary antibodies (FLEX polyclonal guinea pig anti-insulin ready-to-use, DAKO IR00261-2, 1:2; monoclonal rabbit anti-glucagon antibody [EP3070], Abcam ab92517, 1:500) and placed at 37°C in rotation for 7 days. This was followed by six washes of 30 min in PBSGT at RT and a final wash in PBSGT overnight at 4°C. Next, samples were incubated in secondary antibodies: goat anti-rabbit Alexa Fluor 647 conjugated antibody (Life Technologies, 1:500), goat anti-Guinea Pig Alexa Fluor 568 conjugated antibody (Life Technologies, 1:500), diluted in 10 ml PBSGNaT for 5 days at 37°C in a rotating tube. After six 30-min washes in PBS at room temperature, the samples were stored in PBS at 4°C in the dark until clearing. Samples were washed with PBSGNaT (five times, RT) and PBS (five times, RT), dehydrated with a methanol gradient, then washed in 100% methanol (three times, 1 hr each) and incubated overnight with 66% DCM/33% Methanol and then in 100% DCM until the sample sank (15 min to 45 min). The clearing step was performed in 100% Benzyl ether (DBE; Sigma-Aldrich).

#### Light-sheet imaging

3D imaging was performed as previously described (Belle et al., 2014). An ultramicroscope (LaVision BioTec) using ImspectorPro software (LaVision BioTec) was used to perform imaging. The light sheet was generated by a laser (wavelength 488 or 561 nm, Coherent Sapphire Laser, LaVision BioTec) and two cylindrical lenses. A binocular stereomicroscope (MXV10, Olympus) with a 2× objective (MVPLAPO, Olympus) was used at different magnifications (1.6×, 4×, 5×, and 6.3×). Samples were placed in an imaging reservoir made of 100% quartz (LaVision BioTec) filled with DBE and illuminated from the side by the laser light. A PCO Edge SCMOS CCD camera (2560 × 2160 pixel size, LaVision BioTec) was used to acquire images. The step size between each image was fixed at 2 μm.

Images, 3D volume, and movies were generated using Imaris x64 software (version 7.6.1, Bitplane). Stack images were first converted to imaris file (.ims) using ImarisFileConverter and 3D recontruction was performed using “volume rendering”. Optical slices of samples were obtained using the “orthoslicer” tools. The surface of the samples was created using the “surface” tool by creating a mask around each volume. 3D pictures were generated using the “snapshot” tool. Adobe Photoshop CC 2019 (Adobe Systems, San Jose, CA, USA) was used to process, adjust and merge the photomontages. Figures were prepared using Adobe Photoshop CC.

#### Immunohistochemistry on pancreatic sections

Pancreata from control (*n* = 4), PAMH F3 (*n* = 5) and PAMHF3 + SAM (*n* = 5) animals were collected and immersed in 4% paraformaldehyde in PBS for 4 hours. Pancreata were then rinsed in PBS, dehydrated and embedded in paraffin, and the blocks were sectioned on a microtome at 8 μm thickness.

Before immunofluorescence experiments, the sections underwent deparaffinization: incubation in xylene 2 times x 5 min, then rinsed with 100% ethanol (2 times x 5 minutes), and rehydrated in a decreasing gradient of Ethanol/H_2_0 (100%, 90%, 75%, 5 minutes each) and finally transferred to PBS.

Antigen retrieval was performed as follows: the slides were incubated for 30 minutes in 10 mM citrate buffer heated to 95°C. They were brought back to room temperature and rinsed with PBS. The sections were blocked for 30 minutes in blocking buffer (PBS + 7% NGS + 0.3% triton X100), then incubated with primary antibodies in blocking buffer for 48 hours at 4°C. After three rinses in PBS, the sections were incubated for 1h at RT with secondary antibodies diluted in blocking buffer. Finally, the sections were rinsed three times in PBS before nuclear staining with DAPI (1:5000 in PBS) and mounting with Mowiol. The slides were allowed to dry before imaging. Primary antibodies used were: FLEX polyclonal guinea pig anti-insulin ready-to-use (DAKO IR00261-2, 1:2), recombinant monoclonal rabbit anti-glucagon antibody [EP3070] (Abcam ab92517, 1:500). Secondary antibodies: goat anti-rabbit Alexa Fluor 647 conjugated antibody (Life Technologies, 1:500), goat anti-Guinea Pig Alexa Fluor 568 conjugated antibody (Life Technologies, 1:500).

#### Image acquisition and analysis

Single-plane acquisitions of pancreatic sections from control (*n* = 4), PAMH F3 (*n* = 5) and PAMHF3 + SAM (*n* = 5) animals were performed on a Zeiss LSM 710 confocal microscope equipped with a 20X/0.8 objective. Insulin staining was used to measure the area of the pancreatic islets. For each animal, 3 to 4 random regions of the pancreas were imaged, and all pictures were processed in the same manner using Fiji: pictures were first converted to 8bits, and the insulin staining was segmented by manual thresholding with constant parameters (set to 20-255). The area of the segmented signal was then quantified using the measurement tool.

#### RNA extraction and RT-qPCR

Ovaries, perigonadal fat and hypothalami were harvest from control F1 and PAMH F3 (treated or not with SAM) female mice and were snap-frozen in liquid nitrogen. Frozen tissues were homogenized using 1 ml of Trizol (ThermoFisher Scientific, Cat #15596026) with a tissue homogenizer and total RNA was isolated using RNeasy Lipid Tissue Mini Kit (Qiagen; Cat # 74804) following the manufacturer’s instructions. For gene expression analyses, cDNA was synthetized from 1000ng of total RNA using the High Capacity RNA-to-cDNA kit (Applied Biosystems, Cat #4387406) using the manufacturer’s recommended cycling conditions. Real-time PCR was carried out on Applied Biosystems 7900HT Fast Real-Time PCR system using exon-boundary-specific TaqMan Gene Expression Assays (TermoFisher Scientific). Data were analyzed by using the 2^-ΔΔCT^ method (Livak and Schmittgen, 2001) and normalized to housekeeping genes Beta-actin (*ActB*) levels. Values are expressed relative to control values, as appropriate, set at 1.

#### RNA libraries and sequencing

RNA-Seq libraries were generated from 600 ng of total RNA using TruSeq Stranded mRNA Library Prep Kit and TruSeq RNA Single Indexes kits A and B (Illumina, San Diego, CA), according to manufacturer's instructions. Briefly, following purification with poly-T oligo attached magnetic beads, the mRNA was fragmented using divalent cations at 94°C for 2 minutes. The cleaved RNA fragments were copied into first strand cDNA using reverse transcriptase and random primers. Strand specificity was achieved by replacing dTTP with dUTP during second strand cDNA synthesis using DNA Polymerase I and RNase H. Following addition of a single 'A' base and subsequent ligation of the adapter on double stranded cDNA fragments, the products were purified and enriched with PCR (30 sec at 98°C; [10 sec at 98°C, 30 sec at 60°C, 30 sec at 72°C] x 12 cycles; 5 min at 72°C) to create the cDNA library. Surplus PCR primers were further removed by purification using AMPure XP beads (Beckman-Coulter, Villepinte, France) and the final cDNA libraries were checked for quality and quantified using capillary electrophoresis. Libraries were then single-read sequenced with a length of 50 pb, with 8 samples per lane on an Illumina Hiseq4000 sequencer. Image analysis and base calling were carried out using RTA v.2.7.3 and bcl2fastq v.2.17.1.14. Reads were mapped onto the mm10 assembly of Mus musculus genome using STAR (Dobin et al., 2013) v.2.5.3a. Gene expression was quantified from uniquely aligned reads using HTSeq-count (Anders et al., 2015) v.0.6.1p1 with annotations from Ensembl release and union mode. Data quality was evaluated with RSeQC (Wang et al., 2012). Comparisons of read counts were performed using R 3.5.1 with DESeq2 (Love et al., 2014) v1.22.1 Bioconductor package. More precisely, counts were normalized from the estimated size factors using the median ratio method and a Wald test was used for the statistical test. Unwanted variation was identified with sva (Leek, 2014) and considered in the statistical model. To reduce false positive, p-values were adjusted by IHW method (Ignatiadis et al., 2016).

#### MeDIP

MeDIP was performed using MagMeDIP kit (Diagenode) according to the manufacturer’s instructions. Briefly, frozen mouse ovaries (dissected at dioestrus) were chopped and lysed in 1mL GenDNA digestion buffer and DNA was extracted using phenol:chloroform:isoamyl alcohol (25:24:1). DNA extraction of the liver and hypothalamic tissues were performed using the DNeasy Blood & Tissue Kit (Qiagen) according to the manufacturer’s instructions. DNA was quantified using the QubitTM DNA BR Assay kit. 1.1 μg of DNA was sheared by sonication for six cycles with 30 s ON and 30 s OFF at 4 °C using the Bioruptor Plus sonicator (Diagenode). Immunoprecipitation was performed using an anti-5′-methylcytosine mouse monoclonal antibody (Diagenode; Cat nr: C15200081; Lot nr: RD004; 0.2 μg/immunoprecipitation) or a mouse IgG as a negative control (Diagenode; Cat nr: C15400001; Lot nr: MIG002S; 0.2 ug/immunoprecipitation) and magnetic beads, following MagMeDIP kit settings. One-tenth of the DNA sample was set aside at 4 °C for input. To check the efficiency of the MeDIP experiment, spike-in controls including unmethylated (unDNA) and *in vitro* methylated DNA (meDNA) from A. thaliana were used. After magnetic beads washes, methylated DNA was isolated using the DNA Isolation Buffer protocol according to the MagMeDIP kit recommendations. DNA concentration was measured using Qubit dsDNA HS Assay Kit (Thermo Fisher). Efficiency of the immunoprecipitation was assessed by performing qPCR using meDNA and unDNA primers.

MeDIP experiments from human blood were carried using the MagMeDIP protocol as described above with some modifications. DNA was extracted from 200 μL of frozen blood using the QIamp DNA blood Mini kit (Qiagen) according to the manufacturer’s instructions. RNase A was added prior to cell lysis. DNA was eluted in 100 μL of water. Efficiency of the immunoprecipitation of the liver, hypothalamus and human blood samples were assessed by performing qPCR for the mouse/human *TSH2B* (methylated region) and *GAPDH* (unmethylated region) (primers provided in the MagMeDIP kit). Methylation quantification was calculated from qPCR data and reported as the recovery of starting material: % (meDNA-IP/Total input) = 2ˆ[(Ct(10%input)-3.32) − Ct(meDNA-IP)] × 100%.

#### MeDIP-seq - libraries construction and sequencing

Libraries were prepared using the SMART cDNA Library Construction Kit and sequenced on Illumina Hiseq 4000 sequencer as single-end 50 bp reads following Illumina's instructions. Image analysis and base calling were performed using RTA 2.7.3 and bcl2fastq 2.17.1.14. Adapter dimer reads were removed using Dimer Remover. Data were preprocessed with Cutadapt v1.13 (Martin, 2011) to remove the first 9 nucleotides and to remove sequences with a trailing polyT of at least 10 Ts. Cutadapt was used with the following parameters ‘-u 9 -a T(10) --discard-trimmed’. Reads were mapped to the mouse genome (mm10) using Bowtie v1.0.0 (Langmead et al., 2009) with default parameters except for “-p 3 –m 1 --strata --best”. Methylated regions were detected using MACS v1.4.2 (Zhang et al., 2008) with default parameters except for “-g mm -p 1e-3”. Regions were then annotated with the closest genes with Homer v4.9.1 annotatePeaks.pl (Heinz et al., 2010) with Ensembl v90 annotations.

All regions found in at least 2 replicates of the same condition were retained for the detection of differentially methylated regions. They were then combined to get the union of all peaks using the tool Bedtools merge v2.26.0 (Quinlan and Hall, 2010). Read counts were normalized across libraries using the method proposed by (Anders and Huber, 2010). Statistical comparisons of interest were performed using the method proposed by Love et al. (2014) implemented in the DESeq2 v1.22.2 Bioconductor library. p values were adjusted for multiple testing using the Benjamini and Hochberg (1995) method. MA plot and Manhattan plots are been generated using custom R scripts.

#### Methyl donor S-adenosylmethionine (SAM) treatment

Vaginal cytology was analyzed under an inverted microscope to record the specific stage of the estrous cycle. PAMH F3 offspring were injected intraperitoneally (i.p.) daily for 15 days with 200 μL of a solution containing 0.01M phosphate buffered saline (PBS, pH 7.4) or with SAM (50 mg/Kg/day; New England Biolegends, Cat. B9003S). This concentration was chosen based on previous *in vivo* pharmacological studies using the same drug (Li et al., 2012). Estrous cyclicity was analyzed in adult CNTR offspring (prenatally PBS-treated; Group 1, *n* = 5, 6 month-old) for 25 days and in PAMH F3 animals during 10 days before treatment. One group of PAMH F3 animals (Group 2; *n* = 5) was then injected daily with PBS and another group of animals (Group 3; *n* = 5) was injected with SAM for 15 days.

Tail-blood samples were collected for LH and T measurements at diestrus before the beginning of the treatments, at day 10, and at the end of the treatment, at day 25.

### Quantification and statistical analysis

All analyses were performed using Prism 8 (Graphpad Software, San Diego, CA) and assessed for normality (Shapiro–Wilk test and/or D'Agostino & Pearson test) and variance before subsequent analyses by several statistical tests. Sample sizes were chosen according to standard practice in the field. The investigators were not blinded to the group allocation during the experiments. However, analyses were performed by two independent investigators in a blinded fashion. For each experiment, replicates are described in the figure legends. All comparisons between groups, whose distribution was not normal, were performed using Mann-Whitney U test (comparison between two experimental groups) or Kruskal-Wallis test (comparison between three or more experimental groups) followed by a Dunn’s post hoc analysis. For analyses of populations normally distributed, data were compared using an unpaired two-tailed Student’s t test or a one-way ANOVA for multiple comparisons followed by Tukey’s multiple comparison post hoc test. The number of biologically independent experiments, sample size, p values, age and sex of the animals are all indicated in the main text or figure legends. All experimental data are indicated as mean ± SEM or as the 25^th^–75^th^ percentile, line at median. The significance level was set at p < 0.05.

## Supporting Citations

The following reference appears in the Supplemental Information: Schulze Schulze et al. (2012).

## Supplemental information

Document S1. Figures S1–S7 and Tables S1–S3

Data S1. Differential expression analysis between PAMH F3 and Control F1 (CNTR) ovaries, related to Figure 3 The table lists all genes detected on differential expression analysis from RNA-seq experiments.

Data S2. Differential methylation analysis between PAMH F3 and Control (CNTR) ovaries, related to Figure 4 The table lists all regions detected on differential methylation analysis from MeDIP-seq experiments.

Document S2. Article plus Supplemental information
