# Supplementary material for: Polycystic ovary syndrome is transmitted via a transgenerational epigenetic process
Source: Cell Metab. 2021 Mar 2;33(3):513–530.e8. doi: 10.1016/j.cmet.2021.01.004 (PMC7928942; doi:10.1016/j.cmet.2021.01.004)
Supplement: Document S1. Figures S1–S7 and Tables S1–S3 [file mmc1.pdf]

Cell Metabolism, Volume *33*

Supplemental information

Polycystic ovary syndrome is transmitted via a transgenerational epigenetic process

Nour El Houda Mimouni, Isabel Paiva, Anne-Laure Barbotin, Fatima Ezzahra Timzoura, Damien Plassard, Stephanie Le Gras, Gaetan Ternier, Pascal Pigny, Sophie Catteau-Jonard, Virginie Simon, Vincent Prevot, Anne-Laurence Boutillier, and Paolo Giacobini

Polycystic Ovary Syndrome is Transmitted via a Transgenerational Epigenetic Process

Mimouni, Paiva et al.

Supplementary online material

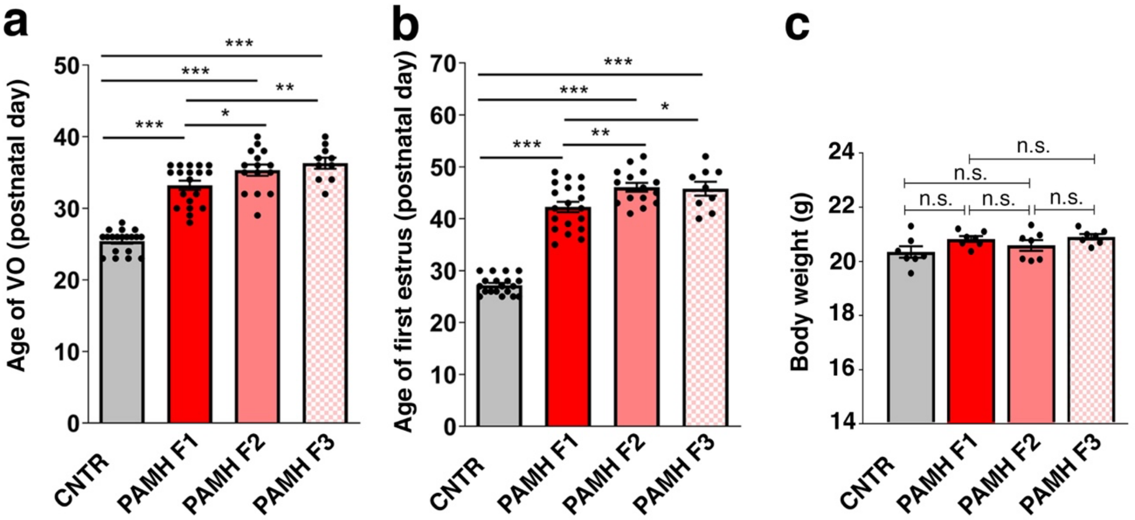

Supplementary Figure 1, related to Fig. 1
Age of puberty onset and body weight in control animals and PAMH lineage.
a, Vaginal opening (VO) in the following prenatally treated offspring: Control (prenatal PBS-treated, n = 19); PAMH F1 (n = 19), F2 (n = 15), F3 (n = 9-10). Differences in time of vaginal opening were assessed by one-way ANOVA ($F_{3,59}$ = 64.64, ****P < 0.0001) and Tukey's multiple comparison *post hoc* test.
b, Age at first estrous in the following prenatally treated offspring: Control (prenatal PBS-treated, n = 19); PAMH F1 (n = 19), F2 (n = 15), F3 (n = 9-10). Differences in time of age of first estrus were assessed by one-way ANOVA ($F_{3,58}$ = 111.7, ****P < 0.0001) and Tukey's multiple comparison *post hoc* test.
c, Body weight in grams (g) of control animals (n = 7) and PAMH F1-F3 (n = 7 each group) adult females (postnatal days (P) 60). Differences in body weight were assessed by one-way ANOVA ($F_{3,24}$ = 2.428, n.s. P = 0.0901) and Tukey's multiple comparison post hoc test.
Data are presented as mean ± s.e.m. Statistical significant was set at P < 0.05, n.s. not significant.

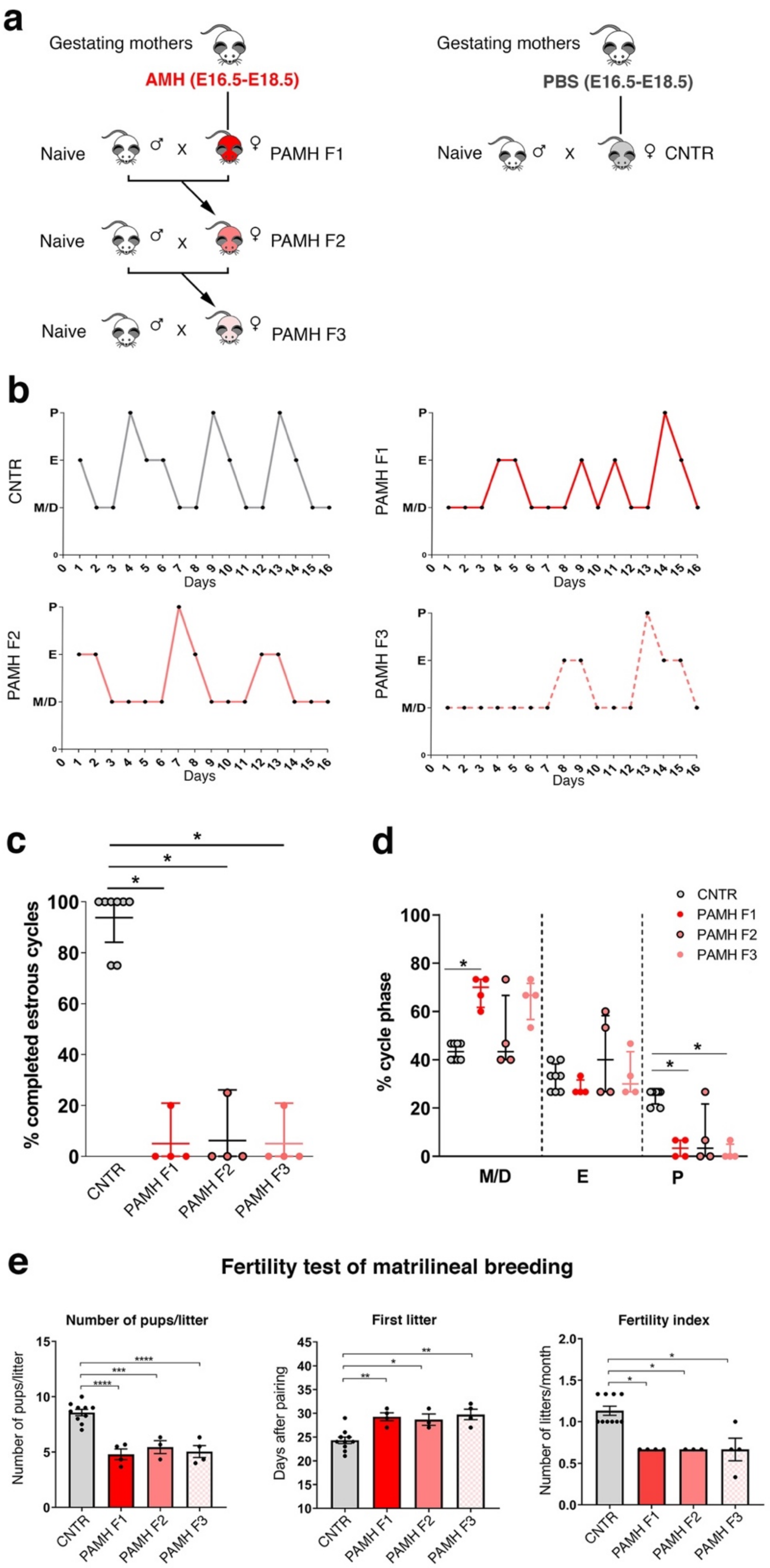
a
Gestating mothers
AMH (E16.5-E18.5)
Naive ♂ x ♀ PAMH F1
Naive ♂ x ♀ PAMH F2
Naive ♂ x ♀ PAMH F3
Gestating mothers
PBS (E16.5-E18.5)
Naive ♂ x ♀ CNTR
b
CNTR
PAMH F1
PAMH F2
PAMH F3
P
E
M/D
Days
c
% completed estrous cycles
CNTR
PAMH F1
PAMH F2
PAMH F3
d
% cycle phase
CNTR
PAMH F1
PAMH F2
PAMH F3
M/D
E
P
e
Fertility test of matrilineal breeding
Number of pups/litter
Number of pups/litter
First litter
Days after pairing
Fertility index
Number of litters/month

Supplementary Figure 2, related to Fig. 1

Ovulatory and fertility alterations in the PAMH lineage occurs via matriline inheritance.

a, Schematic illustration of matrilineal and control breeding scheme.

b, Representative estrous cyclicity of the mice/lineage group during 16 consecutive days. M/D: Metestrus/Diestrus phase, P: Proestrus, E: Estrus.

c, Scatter plot representing the % of completed estrous cycles in the four experimental groups. Quantitative analysis between treatment groups was performed using Kruskal-Wallis test followed by Dunn's post hoc analysis test ($P = 0.0014$). The horizontal line in each scatter plot corresponds to the median value and the vertical line represents the 25th – 75th percentile range.

d, Scatter plot representing the percentage (%) of time spent in each estrous cycle respectively in CNTR ($n = 8$), PAMH F1 females ($n = 4$), PAMH F2 females ($n = 4$), PAMH F3 females ($n = 4$). Comparisons between treatment groups were performed using Kruskal-Wallis test followed by Dunn's multiple comparison *post hoc* test: M/D: * $P = 0.0100$; E: P = 0.5906; P: ** $P = 0.0051$. The horizontal line in each scatter plot corresponds to the median value and the vertical line represents the 25th – 75th percentile range.

e, Fertility was assessed in control and PAMH lineage for 90 days. Fertility tests included the analysis of the number of pups per litter, the delay in days after pairing to give the first litter and the fertility index (number of litters produced per female during 90 days mating protocol). Statistical analysis was performed using one-way ANOVA and Tukey's multiple comparison *post hoc* test: Number of pups/litter, $F_{3,17} = 23.90$ *** $P < 0.0001$; Time to first litter, $F_{3,17} = 9.774$, ***$P = 0.0006$; Analysis of fertility index between groups was assessed using Kruskal Wallis followed by Dunn's multiple comparisons *post hoc* test: **$P = 0.0016$. Data in **e** are represented as mean ± s.e.m. * $P < 0.05$; ** $P < 0.005$; ***$P < 0.0005$; ****$P < 0.0001$.

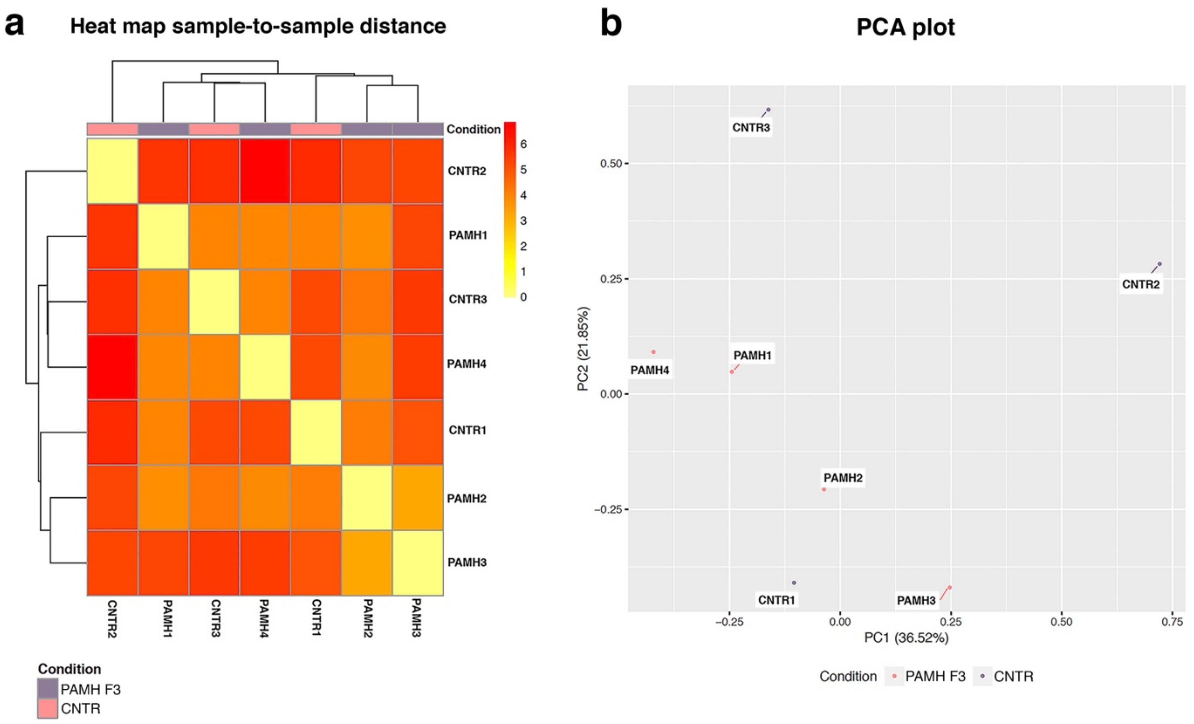

Supplementary Figure 3, related to Fig. 3

Hierarchical clustering and principal component analysis of RNA-seq samples.

a, Heat map depicting hierarchical clustering of sample-to-sample Euclidean distances between the 7 RNA-seq samples (control ovaries, CNTR1-3; PAMH F3 ovaries, PAMH 1-4). Sample-to-sample distances correspond to SERE (Schulze et al., 2012) coefficient. Hierarchical clustering was performed using the Unweighted Pair Group Method with Arithmetic mean (UPGMA) algorithm. Minimum and maximum normalized levels are shown in yellow and red, respectively.

b, Principal component analysis (PCA) plot of the RNA-seq samples. PCi axis represents the principal component i and the number in parenthesis indicates the percentage of explained variance associated with this axis. Principal Component Analysis was computed on regularized logarithm transformed data calculated with the method proposed in (Love et al., 2014).

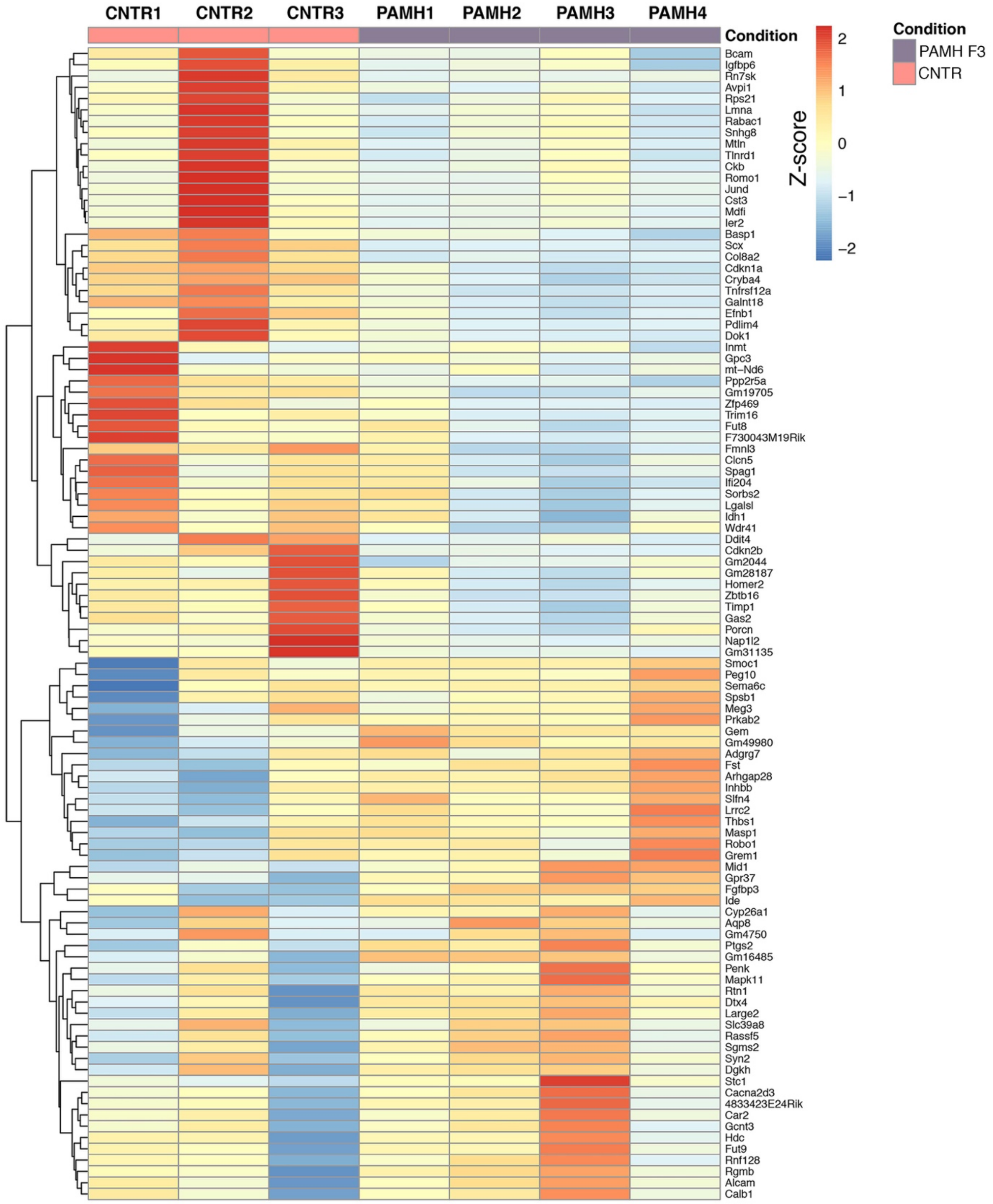

Supplementary Figure 4, related to Fig. 3

Heat map of RNA-seq transcriptome analysis for the 102 DEGs.

Hierarchical clustering was performed using the Unweighted Pair Group Method with Arithmetic Mean (UPGMA) algorithm with the Euclidean distance. Values represented are the normalized read counts divided by transcript length (calculated as the median of the length of all transcripts corresponding to this gene), centered and scaled in the row direction (Z-score).

a **Top 20 upregulated genes by Fold Change (PAMH F3 vs CNTR)**

Gene name	Log2 Fold Change	Biological process
Grem1 *	0,66	negative regulation of BMP signaling pathway; positive regulation of cell population proliferation
Syn2	0,62	neurotransmitter secretion; synaptic vesicle clustering; calcium ion regulated exocytosis
Ide *	0,55	hormone catabolic process; insulin metabolic process; insulin catabolic process
Ptgs2 *	0,5	inflammatory response; prostaglandin biosynthetic process; fatty acid metabolic process
Fgfbp3	0,49	positive regulation of fibroblast growth factor receptor signaling pathway
Stc1	0,48	endothelial cell morphogenesis; embryo implantation
Thbs1 *	0,47	negative regulation of angiogenesis; positive regulation of transforming growth factor beta production
Slfn4	0,46	response to bacterium
Gm16485	0,46	unknown
Gpr37	0,45	G protein-coupled receptor signaling pathway; dopamine biosynthetic process
Cyp26a1	0,44	oxidation-reduction process; retinoic acid catabolic process; retinoic acid metabolic process
Mid1	0,44	positive regulation of stress-activated MAPK cascade; negative regulation of microtubule depolymerization
Masp1	0,44	immune system process; innate immune response; complement activation
Aqp8 *	0,44	transmembrane transport; water transport; positive regulator of apoptosis in granulosa cells
4833423E24Rik	0,44	lipid metabolic process; fatty acid metabolic process; fatty acid biosynthetic process
Fst *	0,44	BMP signaling pathway; female gonad development; negative regulation of activin receptor signaling pathway
Inhbb *	0,43	negative regulation of insulin secretion; activin receptor signaling pathway; SMAD protein signal transduction
Peg10	0,43	cell differentiation; apoptotic process; negative regulation of transforming growth factor beta receptor signaling pathway
Calb1	0,43	calcium ion homeostasis; cytosolic calcium ion concentration
Robo1	0,41	axon guidance; Roundabout signaling pathway; cell migration involved in sprouting angiogenesis

c **RNA-seq validation**

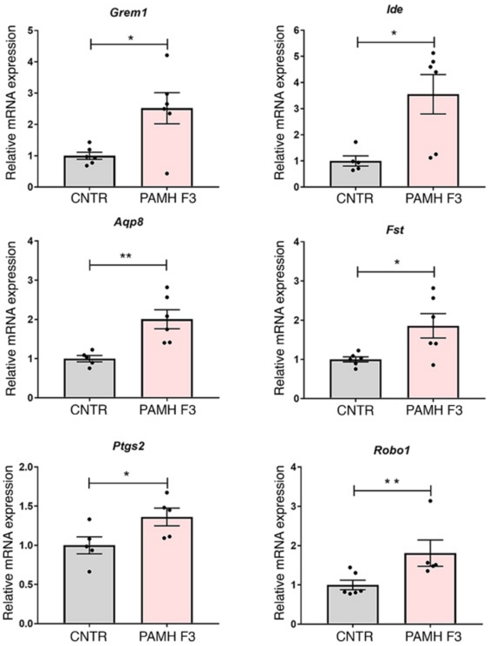

b **Top 20 downregulated genes by Fold Change (PAMH F3 vs CNTR)**

Gene name	Log2 Fold Change	Biological process
Nap1l2	-0,8	positive regulation of histone acetylation
Tnfrsf12a	-0,64	positive regulation of apoptotic process; angiogenesis
Jund	-0,63	regulation of transcription, DNA-templated; regulation of transcription by RNA polymerase II
Cdkn1a *	-0,6	cell cycle arrest; regulation of mitotic cell cycle; signal transduction by p53 class mediator
Rn7sk	-0,59	unknown
Gas2	-0,57	ovulation; initiation of primordial ovarian follicle growth; antral ovarian follicle growth
Romo1	-0,57	defense response to bacterium
Porcn	-0,56	Wnt signaling pathway
Cdkn2b	-0,55	cell cycle; negative regulation of cell population proliferation
Cryba4	-0,55	camera-type eye development; lens development in camera-type eye; visual perception
Scx	-0,55	positive regulation of transcription by RNA polymerase II; BMP signaling pathway
Ier2	-0,54	positive regulation of transcription by RNA polymerase II; cell motility; response to fibroblast growth factor
Wdr41	-0,53	regulation of autophagy
Fmnl3	-0,53	actin cytoskeleton organization; cell migration; angiogenesis
Avpi1	-0,53	activation of MAPK activity; cell cycle
Homer2	-0,52	regulation of G protein-coupled receptor signaling pathway
Inmt	-0,51	methylation; amine metabolic process; response to toxic substance
Zfp469	-0,5	unknown
Gm19705	-0,49	unknown
Gm28187	-0,48	unknown

d

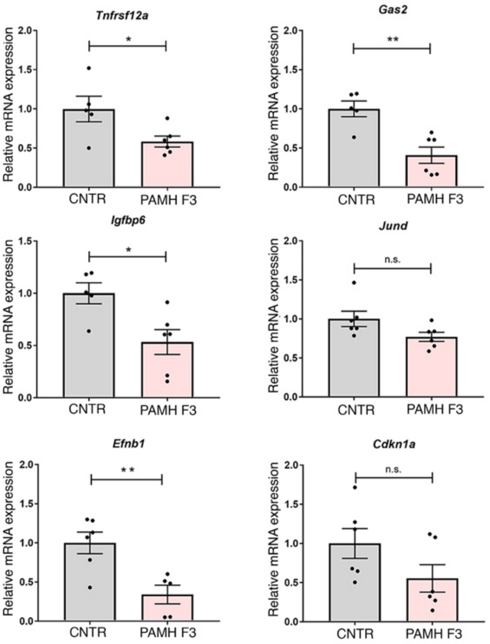

* *Altered expression reported in women with PCOS (see Table S1 for details)*

Supplementary Figure 5, related to Fig. 3

Top 20 upregulated and downregulated differentially expressed genes and RNA-seq validation.

a, List of the top 20 upregulated genes in the third-generation PCOS-like ovaries (PAMH F3) versus control ovaries (CNTR). Asterisks highlight genes whose expression is altered in women with PCOS.

b, List of the top 20 downregulated genes in the third-generation PCOS-like ovaries (PAMH F3) versus control ovaries (CNTR). Asterisk highlights a gene whose expression is altered in women with PCOS.

c, qPCR validation for 6 upregulated genes in CNTR (n = 5-6) and PAMH F3 ovaries (n = 5-6) dissected from adult females (P60) at dioestrus.

d, qPCR validation for 6 downregulated genes in CNTR (n = 5-6) and PAMH F3 ovaries (n = 5-6) dissected from adult females (P60) at dioestrus.

Values are presented as mean ± s.e.m and they are expressed relative to CNTR values, set at 1, after normalization to actin. P value is determined by unpaired two-tailed Student's t test; n.s, not significant; *, **, $P < 0.05$ and $P < 0.005$, compared with the corresponding controls, respectively. Data were combined from two independent experiments.

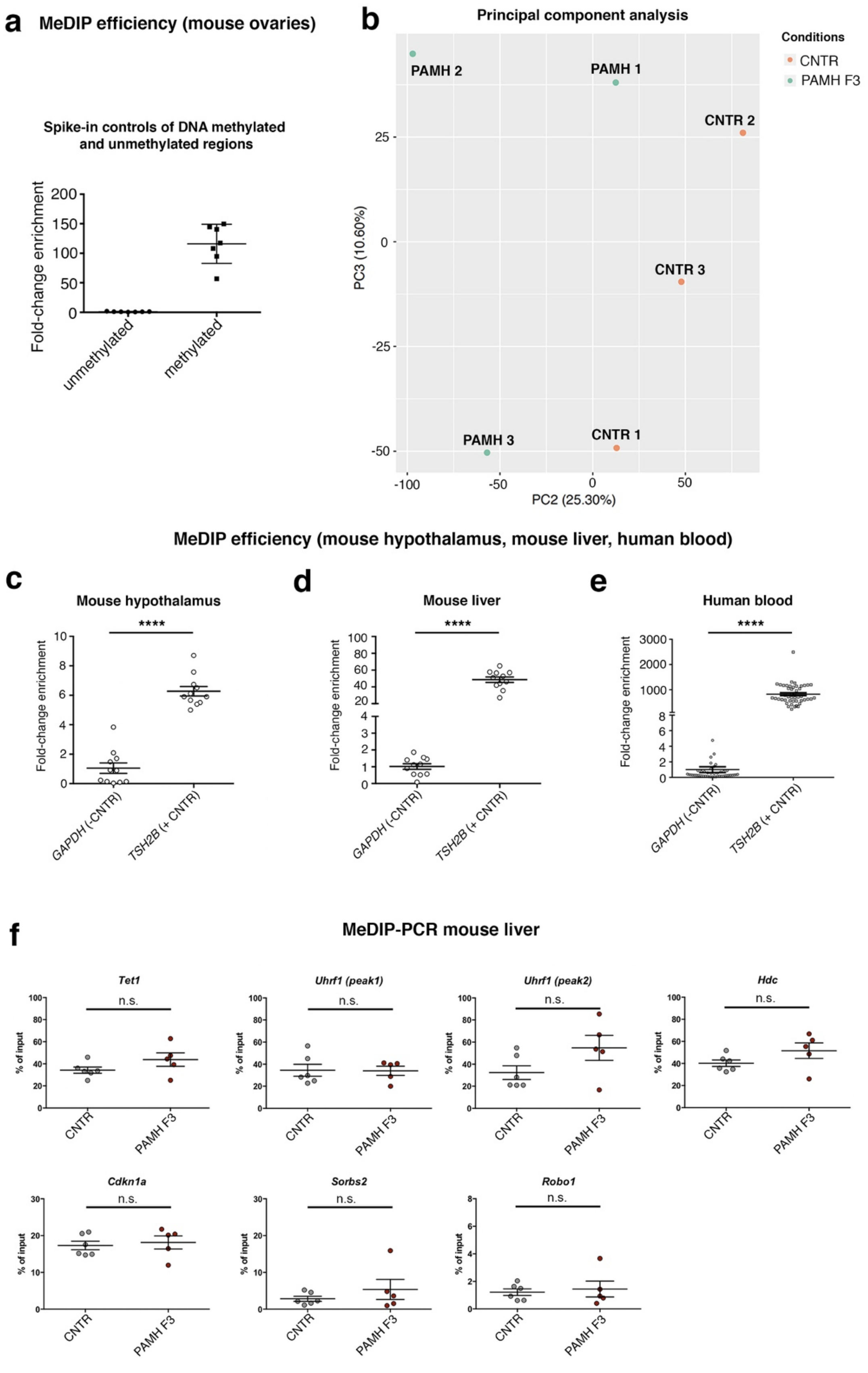

a MeDIP efficiency (mouse ovaries)
Spike-in controls of DNA methylated and unmethylated regions
Fold-change enrichment
unmethylated
methylated
b Principal component analysis
PAMH 2
PAMH 1
CNTR 2
CNTR 3
PAMH 3
CNTR 1
PC3 (10.60%)
PC2 (25.30%)
Conditions
CNTR
PAMH F3
MeDIP efficiency (mouse hypothalamus, mouse liver, human blood)
c Mouse hypothalamus
d Mouse liver
e Human blood
Fold-change enrichment
GAPDH (-CNTR)
TSH2B (+ CNTR)
f MeDIP-PCR mouse liver
Tet1
Uhrf1 (peak1)
Uhrf1 (peak2)
Hdc
Cdkn1a
Sorbs2
Robo1
n.s.
% of input
CNTR
PAMH F3

Supplementary Figure 6, related to Figs. 4 and 5

Technical validations of methylation studies and MeDIP-PCR experiments in the mouse liver.

a, Spike-in controls of DNA methylated and unmethylated regions was used to assess the MeDIP efficiency in mouse ovarian tissues.

b, Principal component analysis (PCA) plot of the MeDIP-seq samples (ovaries). PCi axis represents the principal component i and the number in parenthesis indicates the percentage of explained variance associated with this axis. Principal Component Analysis was computed on regularized logarithm transformed data calculated with the method proposed in (Love et al., 2014). The plot shows the clusters of all replicates of the same conditions, where the MeDIP-seq PAMH samples are very distinguishable from the control samples.

c-**e**, MeDIP efficiency validation in mouse hypothalamus (n = 11; **c**), mouse liver (n = 11; **d**) and human blood samples (n = 47; **e**). MeDIP efficiency was assessed using murine or human primers directed against Glyceraldehyde 3-phosphate dehydrogenase (*GAPDH*), as negative control, and the testicular gene Testis-Specific Histone H2B (*TSH2B*), as a positive control; $P < 0.0001$.

f, MeDIP-PCR experiments in the mouse liver from CNTR (n = 6) and PAMH F3 offspring (n = 5). Data are presented as mean ± s.e.m. Unpaired two-tailed Mann–Whitney U test, significance was set at $P < 0.05$; n.s, not significant.

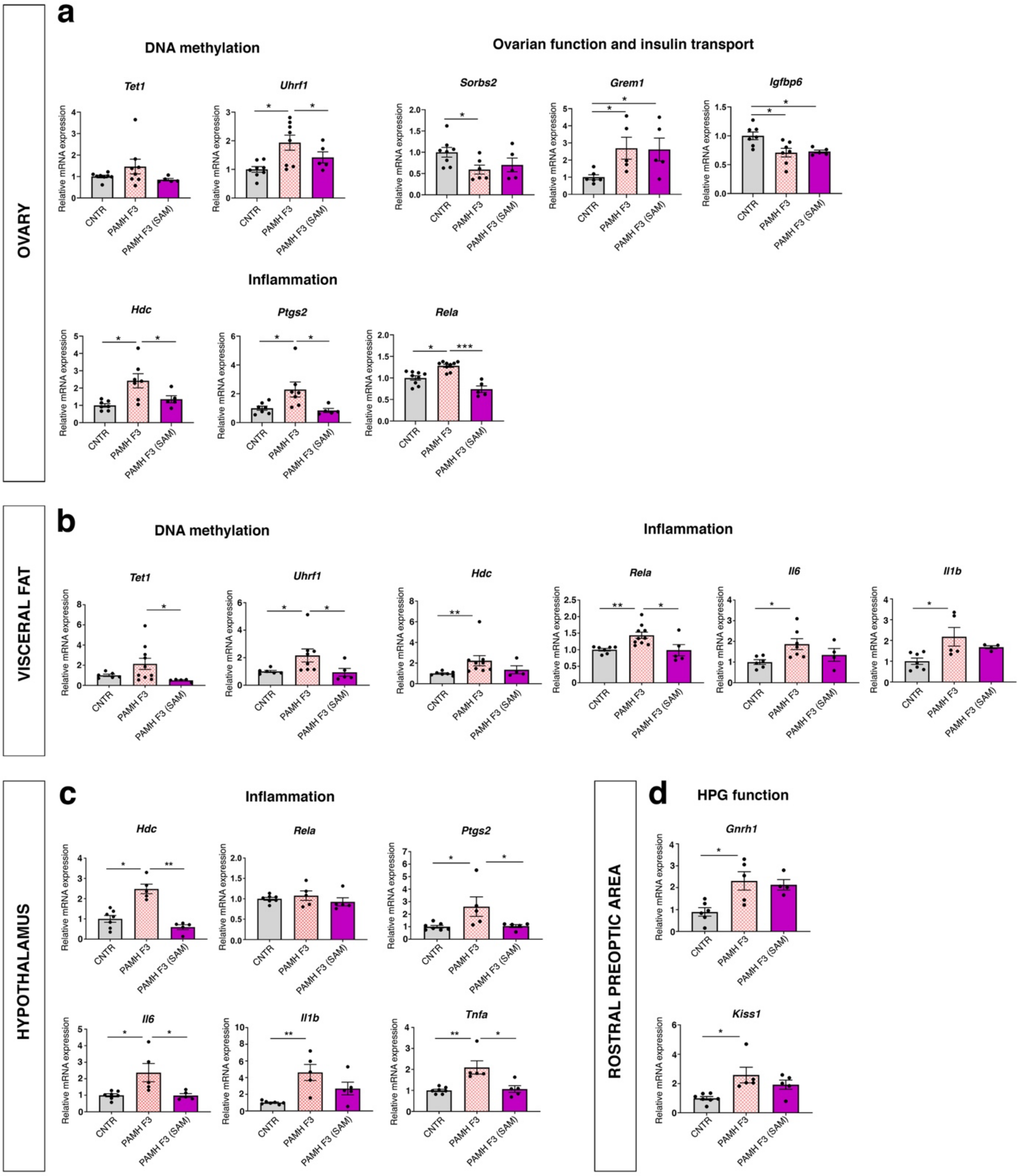
OVARY
a
DNA methylation
Tet1
Uhrf1
Ovarian function and insulin transport
Sorbs2
Grem1
Igfbp6
Inflammation
Hdc
Ptgs2
Rela
VISCERAL FAT
b
DNA methylation
Tet1
Uhrf1
Inflammation
Hdc
Rela
Il6
Il1b
HYPOTHALAMUS
c
Inflammation
Hdc
Rela
Ptgs2
Il6
Il1b
Tnfa
ROSTRAL PREOPTIC AREA
d
HPG function
Gnrh1
Kiss1
Relative mRNA expression
CNTR
PAMH F3
PAMH F3 (SAM)

Supplementary Figure 7, related to Fig. 6

Epigenetic therapy restores expression of genes involved in DNA methylation maintenance and in inflammation in ovarian and metabolic tissues of PAMH F3 offspring.

a, Quantitative RT-PCR analysis of the ovaries harvested from CNTR (n = 6-9, 6 months-old), PAMH F3 (n = 5-9, 6 months-old), PAMH F3-SAM treated (n = 5, 6 months-old) offspring at dioestrus.

b, Quantitative RT-PCR analysis of the perigonadal (visceral) fat harvested from CNTR (n = 6-8, 6 months-old), PAMH F3 (n = 5-10, 6 months-old), PAMH F3-SAM treated (n = 4-5, 6 months-old) offspring at dioestrus.

c, Quantitative RT-PCR analysis of the hypothalamus, comprising the preoptic area and the mediobasal hypothalamus, harvested from CNTR (n = 6-7, 6 months-old), PAMH F3 (n = 5, 6 months-old), PAMH F3-SAM treated (n = 5, 6 months-old) offspring at dioestrus.

d, Quantitative RT-PCR analysis of the preoptic area (POA) dissected from CNTR (n = 6-7, 6 months-old), PAMH F3 (n = 5, 6 months-old), PAMH F3-SAM treated (n = 4-5, 6 months-old) offspring at dioestrus. Values are presented as mean ± s.e.m and they are expressed relative to CNTR values, set at 1, after normalization to actin. Statistical analysis was performed using Kruskal Wallis followed by Dunn's multiple comparisons *post hoc* test; * $P < 0.05$; ** $P < 0.005$; ***$P < 0.0005$.

Supplementary tables

Gene symbol	Gene name	Gene expression in PAMH F3 ovaries vs CNTR	Gene or protein alteration in women with PCOS	Reference
Grem1	Gremlin-1	↑	Single-cell RNAseq cumulus cells ↑ in PCOS	PMID: 28004769
Ide	Insulin-degrading enzyme	↑	Polymorphism associated with metabolic features of PCOS women	PMID: 17953957
Ptgs2	Prostaglandin-endoperoxide synthase 2	↑	Single-cell RNAseq oocytes ↑ in PCOS	PMID: 28004769
Thbs1	Thrombospondin 1	↑	Serum levels ↓ in PCOS	PMID: 26391700
Aqp8	Aquaporin 8	↑	qPCR in ovarian tissue ↑ in PCOS	PMID: 31258711
Fst	Follistatin	↑	Serum levels ↑ in PCOS	PMID: 20093255
Inhbb	Inhibin beta B	↑	Single-cell RNAseq cumulus cells ↑ in PCOS<br>Serum levels ↑ in PCOS	PMID: 28004769<br>PMID: 16720653
Cdkn1a	cyclin-dependent kinase inhibitor 1	↓	Western-blot ovarian cortex ↓ in PCOS	PMID: 25695884

Table S1, related to Fig. 3

Summary of DEGs found in PAMH F3 ovaries (among the top 20 up- or down-regulated genes) which are altered in women with PCOS.

	Control (n = 15)	PCOS (n = 32)	P value
Age (years)	56 ± 3.6	30 ± 2.2	0.0002
BMI (kg/m^2)	22.77 ± 1.1	26.8 ± 1.1	0.1445
Biological hyperandrogenism	n.d.	57.69%	
Clinical hyperandrogenism	13.33%	66.66%	0.002
Irregular cycles	0 %	100 %	< 0.0005
PCO morphology	n.d.	100 %	

Reproductive/ Endocrine measure	Control (n = 15)	PCOS (n = 21-26)	Normal range
Waist circumference	n.d.	85 ± 3.7 (n = 21)	< 80
LH (UI/L)	n.d.	6 ± 1.01 (n = 26)	0.5-6
FSH (UI/L)	n.d.	5.6 ± 0.26 (n = 26)	1-8
T (ng/ml)	n.d.	0.54 ± 0.05 (n = 26)	0.05-0.39
Androstenedione (ng/ml)	n.d.	2.32 ± 0.17 (n = 26)	0.8-2.1
SHBG (nmol/L)	n.d.	38.25 ± 4.15 (n = 24)	30-60
Fasting Insulin (mUI/L)	n.d.	4.45 ± 1.027 (n = 24)	≤ 11
HDL cholesterol (g/L)	n.d.	0.5 ± 0.03 (n = 25)	> 0.5
Triglycerides (g/L)	n.d.	0.72 ± 0.13 (n = 21)	< 1.5
Fasting Glycemia (g/L)	n.d.	0.85 ± 0.01 (n = 23)	< 1.1

Table S2, related to Fig. 7
Clinical characteristics in women with and without PCOS.
Data are median ± range. Comparisons between groups were made using the Mann-Whitney test. Clinical hyperandrogenism was defined by the presence of hirsutism (modified Ferriman-Gallwey score over 7 and/or acne located in more than two areas). Irregular cycles were defined as a menstrual cycle > 35 days. Abbreviations: PCOS: polycystic ovary syndrome; BMI: Body mass index; SHBG: sex hormone binding globulin; LH: luteinizing hormone; FSH: follicle stimulating hormone; T: testosterone; HDL: high density lipoprotein.

	Control (n = 3)	PCOS (n = 5)	P value
Age (years)	24 ± 4.4 (n = 3)	26 ± 2.01 (n = 5)	> 0.99
BMI (kg/m^2)	21.15 ± 0.01 (n = 2)	24.88 ± 3.5 (n = 5)	
Biological hyperandrogenism	n.d.	60%	
Clinical hyperandrogenism	33.33%	33.33%	1
Irregular cycles	0 %	100 %	< 0.0005
PCO morphology	n.d.	100 %	

Reproductive/ Endocrine measure	Control (n = 3)	PCOS (n = 3-5)	Normal range
Waist circumference	n.d.	78 ± 6.0 (n = 5)	< 80
LH (UI/L)	n.d.	14.8 ± 2.7 (n = 5)	0.5-6
FSH (UI/L)	n.d.	5.8 ± 0.5 (n = 5)	1-8
T (ng/ml)	n.d.	0.35 ± 0.1 (n = 5)	0.05-0.39
Androstenedione (ng/ml)	n.d.	2.32 ± 0.28 (n = 5)	0.8-2.1
SHBG (nmol/L)	n.d.	30.95 ± 3.5 (n = 4)	30-60
Fasting Insulin (mUI/L)	n.d.	8.45 ± 1.5 (n = 4)	≤ 11
HDL cholesterol (g/L)	n.d.	0.47 ± 0.07 (n = 5)	> 0.5
Triglycerides (g/L)	n.d.	0.77 ± 0.3 (n = 3)	< 1.5
Fasting glycemia (g/L)	n.d.	0.85 ± 0.02 (n = 4)	< 1.1

Table S3, related to Fig. 7

Clinical characteristics of daughters from women with or without PCOS.

Data are median ± range. Comparisons between groups were made using the Mann-Whitney test. Clinical hyperandrogenism was defined by the presence of hirsutism (modified Ferriman-Gallwey score over 7 and/or acne located in more than two areas). Irregular cycles were defined as a menstrual cycle > 35 days. Abbreviations: PCOS: polycystic ovary syndrome; BMI: Body mass index; SHBG: sex hormone binding globulin; LH: luteinizing hormone; FSH: follicle stimulating hormone; T: testosterone; HDL: high density lipoprotein.
